# Genome-Wide Identification of the Ca^2+^-ATPase Gene Family and Functional Analysis of *MdACA39* in Resistance to *Alternaria alternata* in *Malus domestica*

**DOI:** 10.3390/plants15162421

**Published:** 2026-08-08

**Authors:** Yingjun Hou, Mingzhi Guan, Wenhui Wang, Wenfang Li, Zonghuan Ma, Xin Li, Cunwu Zuo, Juan Mao, Baihong Chen

**Affiliations:** College of Horticulture, Gansu Agricultural University, Lanzhou 730070, China; houyj@gsau.edu.cn (Y.H.); gmz457562@163.com (M.G.); wwh18894208396@126.com (W.W.); liwenf@gsau.edu.cn (W.L.); mazh@gsau.edu.cn (Z.M.); lixin@gsau.edu.cn (X.L.); zuocw@gsau.edu.cn (C.Z.); maojuan@gsau.edu.cn (J.M.)

**Keywords:** *Malus domestica*, Ca^2+^-ATPase gene family, *MdACA39*, *A. alternata*, transient expression

## Abstract

The calcium ion-transporting ATPase (Ca^2+^-ATPase) gene family maintains plant intracellular Ca^2+^ homeostasis and regulates growth, development and stress immunity; however, its functions remain poorly characterized in *Malus domestica*. Here, we performed a genome-wide identification of apple Ca^2+^-ATPase genes and obtained 45 members, which were classified into *MdACA* (39) and *MdECA* (6) subfamilies and unevenly distributed on 14 chromosomes. Phylogenetic analysis of Ca^2+^-ATPase genes from *Malus domestica, Arabidopsis thaliana,* and *Oryza sativa* classified these proteins into five subgroups. The ACA and ECA subfamilies were highly conserved across species, whereas Group D was apple-specific. Collinearity and Ka/Ks analyses indicated that segmental duplication and purifying selection dominated the evolution of apple Ca^2+^-ATPase genes. Promoter *cis*-element prediction uncovered numerous regulatory elements related to phytohormone signaling, growth, development and stress defense. Codon usage bias analysis indicated that AUG (methionine) was the dominant codon. Tissue expression profiles showed differential expression of apple Ca^2+^-ATPase genes in various organs. Quantitative real-time PCR (qRT-PCR) assays demonstrated widespread responses of Ca^2+^-ATPase genes to *Alternaria alternata* infection, exogenous CaCl_2_, salicylic acid (SA) and methyl jasmonate (MeJA), among which *MdACA39* was strongly induced under all treatments. Subcellular localization verified that MdACA39 resides on the plasma membrane. Moreover, transient overexpression of *MdACA39* significantly enhanced apple resistance to *A. alternata*, likely due to the activation of SA, MeJA and Ca^2+^ signaling-mediated immune pathways, the induction of disease resistance-related genes, and elevated antioxidant enzyme activity. Collectively, this study systematically characterizes the apple Ca^2+^-ATPase family and identifies *MdACA39* as a key regulator of fungal resistance, providing valuable gene resources for dissecting Ca^2+^ signaling-mediated disease resistance in apple.

## 1. Introduction

Apple is one of the most extensively cultivated and economically significant deciduous fruit tree species worldwide, occupying a pivotal position in the commercial fruit industry. In practical apple production, plants frequently suffer from diverse biotic stresses and physiological disorders. Notably, apple blotch leaf spot disease, caused by the fungal pathogen *A. alternata* f. sp. mali, is prevalent in major apple-producing regions of China. This disease severely impairs apple yield and fruit quality, restricting the sustainable and high-quality development of the apple industry [1]. Calcium is an indispensable mineral nutrient for the growth and development of apple trees. Calcium ions (Ca^2+^), acting as pivotal second messengers in plant cells, extensively modulate plant growth, developmental processes, and responses to internal and external environmental stimuli. They play a particularly essential role in activating immune regulatory pathways during pathogen invasion [2]. Plant-pathogen interactions constitute a dynamic and sophisticated immune regulatory network. Plants recognize pathogen-associated molecular patterns (PAMPs), such as fungal chitin, via plasma membrane-localized pattern recognition receptors (PRRs), thereby triggering the primary immune defense mechanism known as pattern-triggered immunity (PTI). The earliest physiological event triggered upon PAMP perception is the generation of Ca^2+^ oscillation signatures, which further activate downstream calcium sensor proteins. This signaling cascade initiates multilayered plant defense responses, including reactive oxygen species (ROS) bursts, cell wall callose deposition, SA and JA biosynthesis, hypersensitive response (HR)-mediated cell death, and the upregulation of pathogenesis-related (PR) genes. This series of physiological and molecular events constitutes the core initiation mechanism of the plant PTI signaling pathway [3,4,5]. To overcome the PTI-mediated defense barrier, pathogenic fungi secrete effector proteins into host cells to suppress PTI activation. In response, plants have evolved intracellular nucleotide-binding leucine-rich repeat receptors (NLRs) that specifically recognize these effectors and activate a more robust secondary immune response known as effector-triggered immunity (ETI), which is typically accompanied by pronounced HR-associated necrotic lesions [6,7]. Cumulative research evidence confirms that precise spatiotemporal control of cytosolic Ca^2+^ concentration is indispensable for plant resistance to pathogen infection [2]. Consequently, the precise modulation of Ca^2+^ signaling, including its generation, decoding, and signal termination, is fundamental to plant resistance against pathogen infection.

Calcium ion-transporting ATPases (Ca^2+^-ATPases) are essential functional proteins that maintain intracellular Ca^2+^ homeostasis by actively transporting cytosolic Ca^2+^ either out of the cell or into intracellular organelles for sequestration. This process precisely modulates specific calcium signaling patterns during pathogen challenges [8]. Ca^2+^-ATPases belong to the P-type ATPase superfamily and are divided into two major subfamilies based on structural characteristics and subcellular localization: type IIA endoplasmic reticulum (ER)-type calcium pumps (ECAs), predominantly localized to the ER; and type IIB autoinhibited calcium pumps (ACAs), which are widely distributed across the plasma membrane, ER, and tonoplast [9]. Notable structural and functional differences exist between these two subfamilies. ACA members contain an N-terminal autoinhibitory domain responsible for calmodulin (CaM) binding, which is absent in ECA proteins. Additionally, ECA pumps exhibit strict substrate specificity for ATP, whereas membrane-localized ACAs can hydrolyze multiple nucleotide substrates, including GTP and ITP [10,11]. To date, Ca^2+^-ATPase gene families have been systematically identified and characterized in various plant species, including *Arabidopsis*, rice, wheat, sugar beet, pepper, and banana [12,13,14,15,16]. Previous studies have revealed significant interspecies variation in the number of Ca^2+^-ATPase family members, with ACA genes generally outnumbering ECA genes, and an uneven chromosomal distribution of family members.

The plant Ca^2+^-ATPase gene family plays a crucial role in regulating plant growth, development, and responses to biotic stress. Current research has primarily focused on the ACA subfamily, while the biological functions of the ECA subfamily remain poorly understood. In *Arabidopsis thaliana*, the ER-localized *AtECA1* and *AtECA3* exhibit functional redundancy; their double mutants display severe developmental retardation, leaf tip necrosis, and chlorosis. Plasma membrane-localized *ataca4* and *ataca11* double mutants show significantly reduced rates of new leaf growth [17,18]. *AtACA9* and *AtACA11* are highly expressed during pollen development, and knockout of *ataca9* results in male sterility in *Arabidopsis* [19]. The *ataca1/2/7* triple mutant exhibits damaged leaf morphology and reduced leaf size [18]. These findings indicate that distinct Ca^2+^-ATPase members coordinate to regulate the development and normal growth of plant vegetative organs through subcellular compartment-specific functional differentiation. The ACA subfamily exerts a dual regulatory effect on plant biotic stress responses via complex functional mechanisms. In *Arabidopsis*, transcription of *AtACA12* and *AtACA13* is maintained at low levels under normal growth conditions but is markedly induced following pathogen infection [20]. In rice, *OsACA5* expression is significantly upregulated after infection with *Magnaporthe oryzae* or treatment with PAMPs such as flg22 [21]. In wheat, multiple Ca^2+^-ATPase genes are transcriptionally activated in response to *Fusarium graminearum* infection [22]. The plasma membrane-resident *AtACA8* and *AtACA10* function as core Ca^2+^ pumps that assemble signaling complexes with the flagellin receptor FLS2 to positively modulate disease resistance. The *aca8 aca10* double mutant exhibits markedly attenuated early Ca^2+^ influx, ROS production, and downstream defense gene expression upon flg22 treatment, ultimately leading to compromised resistance to bacterial pathogens [23,24]. Conversely, tonoplast-localized AtACA4 and AtACA11 negatively regulate PTI signaling; mutations in these genes enhance cytosolic Ca^2+^ signaling and ROS bursts, thereby improving plant disease resistance but simultaneously causing growth inhibition and premature leaf senescence [25,26,27]. Consistent dual functions of ACA genes in the growth-defense trade-off are also observed in rice. Silencing *OsACA9* enhances resistance to *Magnaporthe oryzae* and *Xanthomonas oryzae* but induces premature leaf senescence and wilting; *osaca5* mutants display improved *Magnaporthe oryzae* resistance accompanied by reduced plant height and seed-setting rate [21,27]. Collectively, ACA genes participate in immune regulation by shaping specific calcium signals and mediate the trade-off between growth and defense, demonstrating remarkable functional complexity and diversity.

Current research concerning plant Ca^2+^-ATPase gene families has largely focused on bioinformatic characterization in model plants including *Arabidopsis thaliana* and rice, as well as crops such as wheat and banana. Most published studies remain confined to genome-wide identification and evolutionary analysis, while in-depth functional verification of candidate genes involved in disease resistance is relatively scarce. To date, no systematic study integrating evolutionary characterization, stress-induced expression profiling, and in vivo functional verification of the Ca^2+^-ATPase gene family has been published in *Malus domestica*. Herein, we performed genome-wide identification and comprehensive bioinformatic analysis of apple Ca^2+^-ATPases, combined with tissue-specific expression detection and multi-stress expression screening. Critically, we further characterized the immune function of *MdACA39* against *A. alternata*. Beyond routine gene family characterization, this work provides new experimental evidence linking Ca^2+^-ATPases to apple fungal disease resistance. Our findings offer insights into calcium transporter-associated stress responses and supply candidate genetic resources for further research on disease resistance breeding in apple.

## 2. Results

### 2.1. Identification and Physicochemical Characterization of the Apple Ca^2+^-ATPase Gene Family

**Figure 1:**
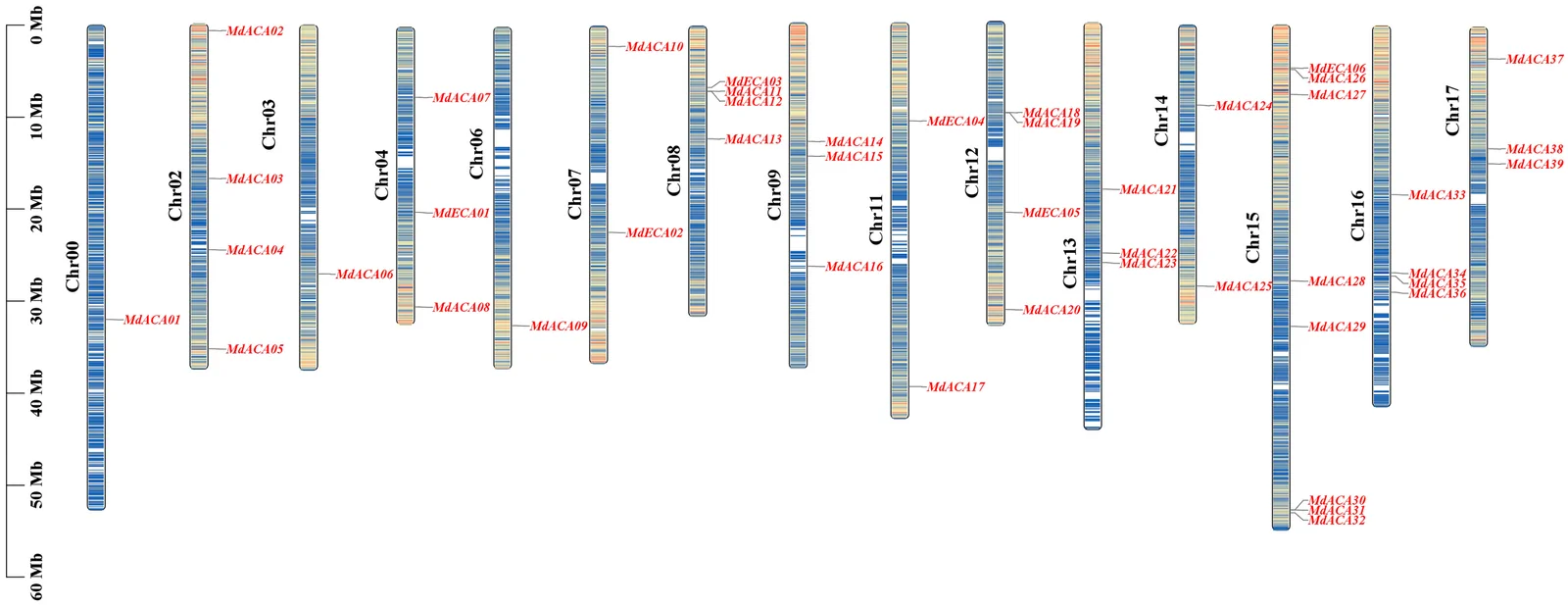
Chromosomal distribution of the Ca^2+^-ATPase gene family members in apple. The varying colors along the chromosomes correspond to gene density levels. The scale on the left denotes chromosome length in megabases (Mb).

In this study, a total of 45 Ca^2+^-ATPase family genes were systematically identified within the complete apple genome (Figure 1). Based on the presence or absence of an autoinhibitory domain at the N-terminus of their protein sequences, these genes were classified into two distinct subfamilies: ACA and ECA. The ACA subfamily contained 39 members designated *MdACA01*–*MdACA39*, while the ECA subfamily contained 6 members named *MdECA01*–*MdECA06*. Chromosomal localization analysis revealed that most Ca^2+^-ATPase genes were unevenly distributed across 14 apple chromosomes, with only *MdACA1* assigned to unclassified scaffolds (Chr00), and chromosome 15 harboring the greatest number (eight genes). On this chromosome, *MdECA06* is physically proximal to *MdACA26*; *MdACA30* and *MdACA31* are closely linked to *MdACA32*. Similarly, chromosomes 8 (*MdECA03*, *MdACA11* and *MdACA12*), 12 (*MdACA18* and *MdACA19*), and 16 (*MdACA34* and *MdACA35*) exhibit adjacent gene arrangements, suggesting potential genetic linkage among these gene pairs.

Physicochemical property analyses (Table S1) showed that the amino acid lengths of apple Ca^2+^-ATPases proteins range from 485 (MdACA06) to 2062 (MdACA33) aa, with molecular weights varying from 52.43 to 224.01 kDa. The theoretical isoelectric points (pI) range from 5.31 to 8.55, with ten proteins classified as basic and the remainder as acidic. The instability index spans from 29.77 to 40.36, identifying MdACA05, MdACA27, and MdECA03 as unstable proteins, while the others are stable. The aliphatic index ranges from 94.75 to 110.19, and the grand average of hydropathicity (GRAVY) varies from −0.050 to 0.231. Except for MdACA17 and MdACA19, which are hydrophilic, all other proteins are hydrophobic.

### 2.2. Prediction of Subcellular Localization and Secondary Structure Analysis of Apple Ca^2+^-ATPase Proteins

Predictions of subcellular localization indicate that apple Ca^2+^-ATPase proteins (Table S2) predominantly localize to the plasma membrane, endoplasmic reticulum, and vacuoles, with the plasma membrane exhibiting the highest localization scores. Notably, MdACA21 is exclusively abundant at the plasma membrane and absent from other organelles.

Secondary structure analysis revealed that all family members (Table S3) contain four canonical structural elements: α-helix, β-turn, random coil, and extended strand, with considerable variation in their relative proportions. The α-helix (40.09–50.79%) and random coil (27.77–33.00%) constitute the primary structural components, followed by extended strands (15.31–22.60%), while β-turns represent the smallest fraction (4.82–7.59%).

### 2.3. Phylogenetic Analysis of Ca^2+^-ATPase Gene Family Members in Apple and Representative Plant Species

**Figure 2:**
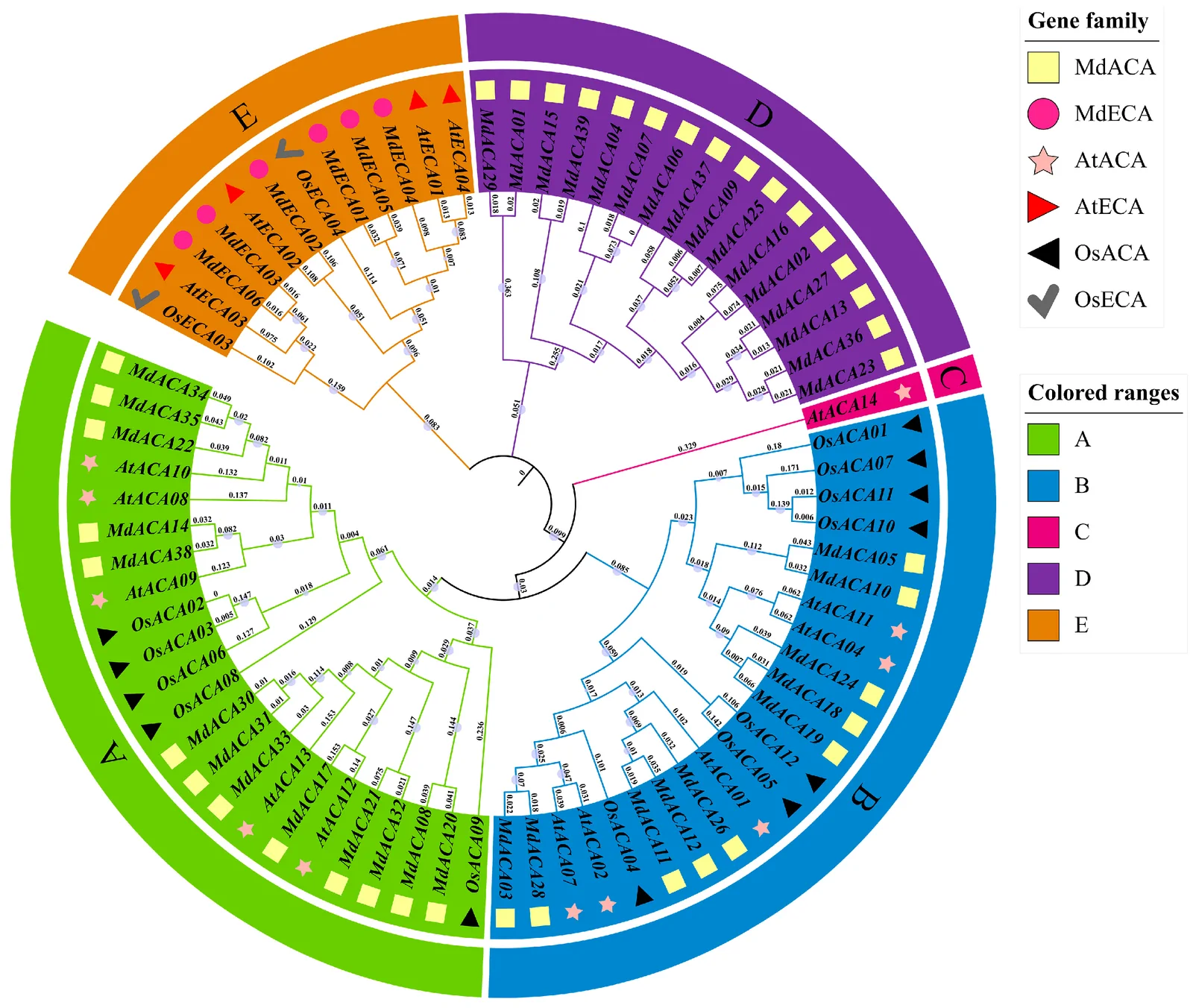
Phylogenetic analysis of Ca^2+^-ATPase proteins in apple (Md), *Arabidopsis* (At) and rice (Os). The phylogenetic tree was constructed using the Neighbor-Joining method in MEGA version 11.0, with evolutionary distances calculated by the *p*-distance method. Distinct clades are indicated by different colors: Group A (light green), Group B (blue), Group C (pink), Group D (light purple), Group E (brownish yellow). Species are denoted by specific-colored symbols as follows: apple (ACA yellow squares and ECA pink circles), *Arabidopsis* (ACA light pink stars and ECA red triangles), rice (ACA black triangles and ECA gray checkmarks).

To investigate the evolutionary relationships of Ca^2+^-ATPases across different plant species, a phylogenetic tree (Figure 2) was constructed using Ca^2+^-ATPase protein sequences from apple, *Arabidopsis*, and rice. The phylogenetic analysis classified these proteins into five distinct subgroups (Groups A–E). Groups A, B, C, and D correspond to the ACA subfamily, while Group E aligns with the ECA subfamily. Notably, Group D comprises only apple ACA proteins, suggesting an apple-specific expansion of this subgroup. The ACA and ECA subfamilies were clearly separated, indicating functional divergence. Moreover, the apple ACA proteins were intermingled with those from *Arabidopsis* and rice in Groups A, B, and C, suggesting that these subgroups are evolutionarily conserved across monocots and dicots. Group C solely contains the single gene *AtACA14* from *Arabidopsis thaliana*, implying that it has undergone distinct sequence divergence during evolution. These results demonstrate that the apple Ca^2+^-ATPase family has undergone both conserved evolution and lineage-specific expansion.

### 2.4. Analysis of Collinearity and Gene Duplication Within the Apple Ca^2+^-ATPase Gene Family

**Figure 3:**
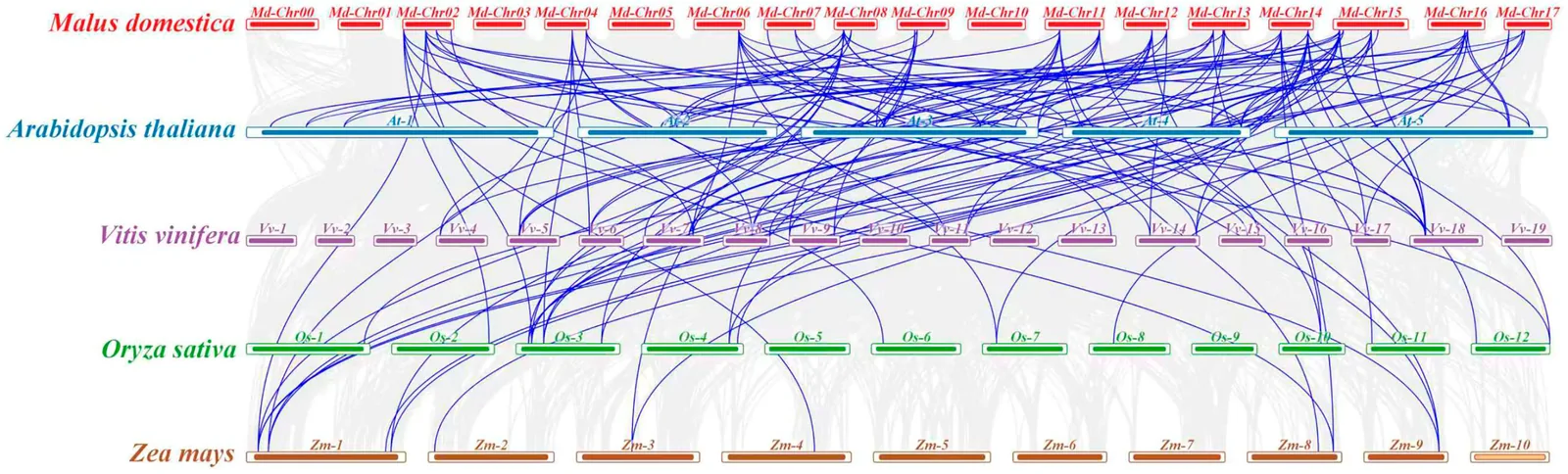
Collinearity analysis of Ca^2+^-ATPase genes among *Arabidopsis thaliana*, grape, rice, maize, and apple. The gray lines in the background denote collinear genomic blocks among the genomes of apple, maize, *Arabidopsis*, rice, and grape, whereas the blue lines emphasize the syntenic PERK gene pairs.

To understand the expansion mechanisms of the apple Ca^2+^-ATPase gene family, we performed inter- and intra-species collinearity analyses. Interspecies collinearity analysis of the Ca^2+^-ATPase gene families among apple, maize, *Arabidopsis*, grape, and rice is presented in Figure 3, revealing extensive gene duplication events and numerous homologous collinear gene pairs. Apple shares more homologous pairs with the dicotyledons *Arabidopsis* and grape (45 and 44 pairs, respectively) than with the monocotyledons rice and maize (26 and 20 pairs, respectively), totaling 135 cross-species homologous gene pairs. These results confirm closer evolutionary relationships among dicots.

**Figure 4:**
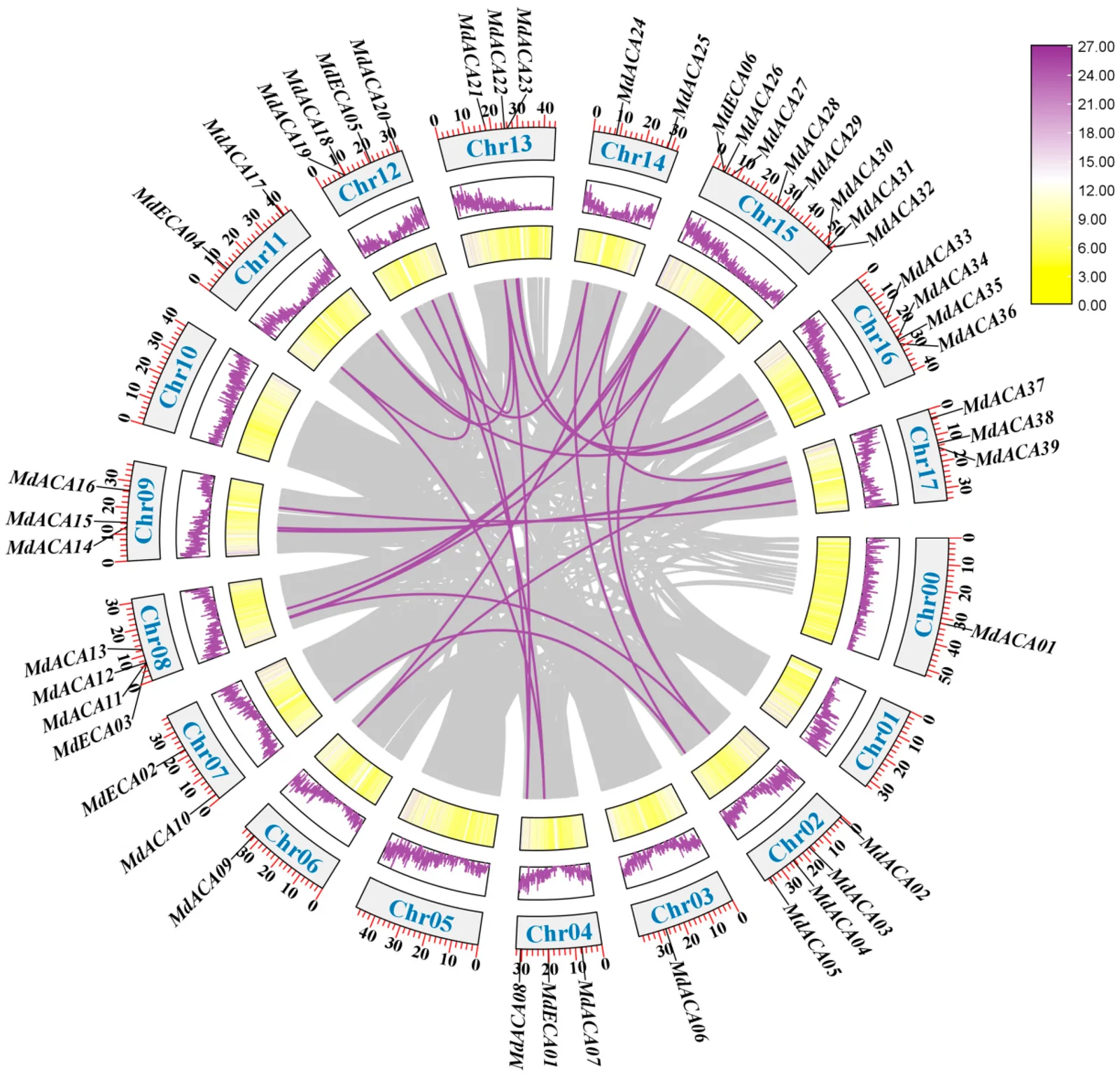
Collinearity analysis of the Ca^2+^-ATPase gene family in apple. The purple lines denote duplicated pairs of Ca^2+^-ATPase genes.

Intraspecies collinearity analysis within apple identified 25 duplicated gene pairs among the 45 Ca^2+^-ATPase genes (Figure 4). No tandem duplications on the same chromosome were observed, indicating that segmental duplications primarily drove the expansion of this gene family. The Ka/Ks ratio calculation revealed that all duplicated gene pairs exhibited Ka/Ks values < 1 (Figure S1), suggesting that the apple Ca^2+^-ATPase genes have undergone strong purifying selection during evolution, with limited functional divergence after duplication.

### 2.5. Analysis of Conserved Motifs, Promoter Cis-Acting Elements, and Gene Structure in Apple Ca^2+^-ATPase Genes

**Figure 5:**
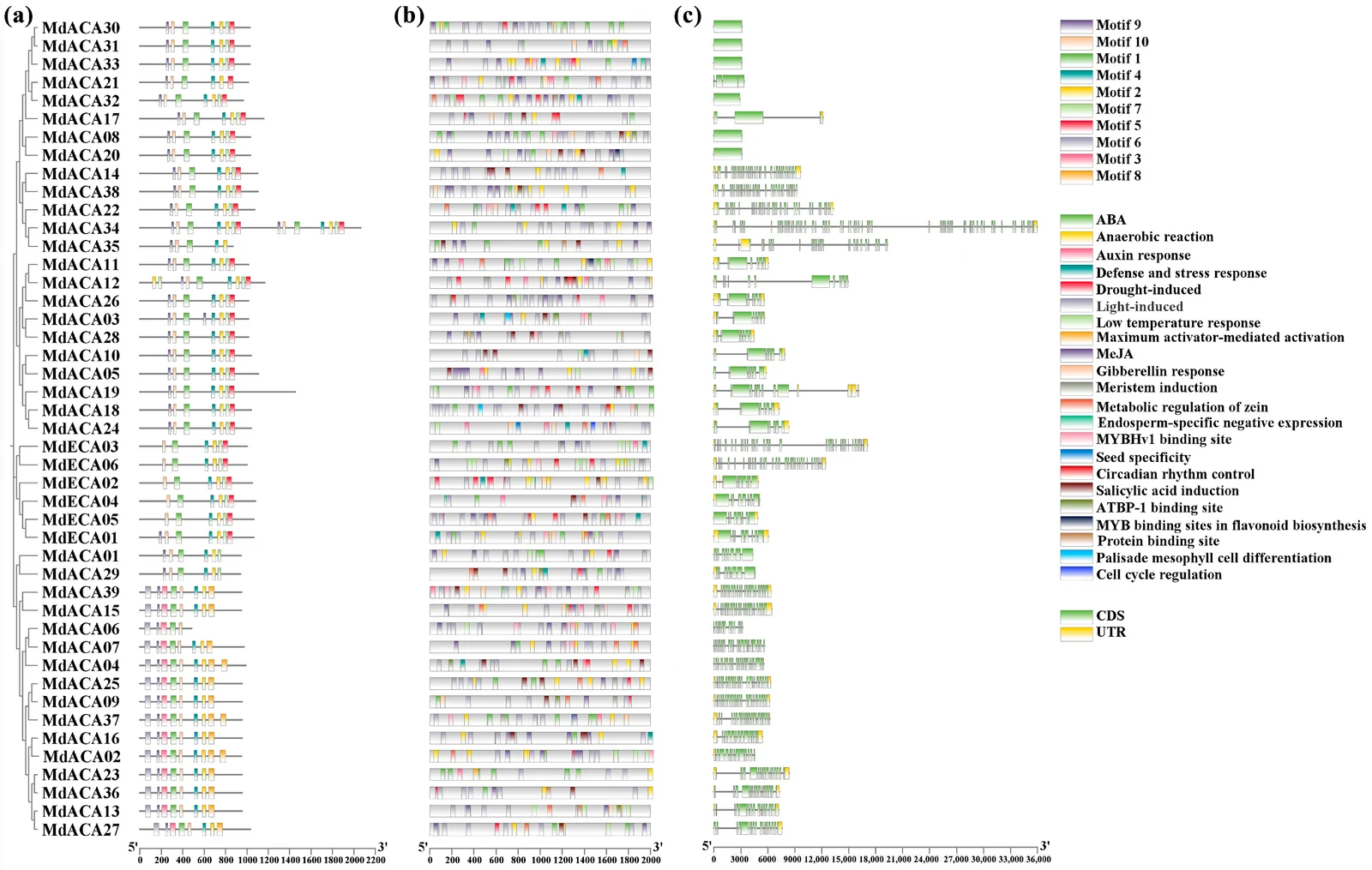
Analysis of motifs, characterization of promoter *cis*-acting elements and gene structure within the apple Ca^2+^-ATPase gene family. (**a**) Identification and distribution of conserved motifs in apple Ca^2+^-ATPase proteins. (**b**) Examination of *cis*-acting elements located within the 2000 bp upstream promoter regions of apple Ca^2+^-ATPase genes. (**c**) Intron-exon structure of apple Ca^2+^-ATPase genes.

To gain insight into the structural diversity of the apple Ca^2+^-ATPase family, we analyzed conserved motifs, promoter cis-elements, and gene structures. Motif analysis of the apple Ca^2+^-ATPase proteins revealed the presence of ten distinct motifs (Figure 5a; Figure S2). Notably, all genes consistently contained motifs 1, 2, and 10, and all genes contained motif 4 (Except for MdACA06), indicating substantial structural conservation and functional similarity. Members of subgroup B exhibit highly consistent motif numbers and arrangements, containing 7–9 conserved motifs in a specific order, reflecting strong structural stability. Promoter analysis (Figure 5b and Figure S3) revealed a widespread presence of *cis*-acting elements responsive to light, hormones, and abiotic stresses across all 45 genes. Every gene contains light-responsive elements, while all except *MdECA04* harbor hormone-responsive elements. MeJA-, ABA-, and SA-responsive elements were identified in 39, 37, and 24 genes, respectively; 37 genes contain anaerobic induction elements; 21 genes possess defense and low-temperature response elements; and 23 genes have drought-responsive elements. Notably, *MdACA01*, *MdACA30*, *MdACA32*, and *MdECA02* are enriched with six major element types: light, hormone, drought, low temperature, anaerobic, and defense, indicating their potential involvement in the multifaceted regulation of apple growth, development, and stress responses. Gene structure analysis (Figure 5c) revealed exon counts ranging from 1 to 67, with *MdACA34* having the highest number. Several genes lack untranslated regions (UTRs): *MdACA05*, *MdACA27*, and *MdECA05* lack 5′ UTRs; *MdACA01*, *MdACA12*, *MdACA35*, and *MdACA38* lack 3′ UTRs; and *MdACA08*, *MdACA20*, *MdACA30*, *MdACA31*, *MdACA32*, *MdACA33*, and *MdECA03* have the fewest exons and lack both 3′ and 5′ UTRs. These findings suggest that exon number, sequence length, and coding region distribution vary within subfamilies, indicating that structural diversity may underlie functional differentiation. The absence of UTRs in some genes may contribute to diversification in transcriptional regulation.

### 2.6. Analysis of Codon Usage Bias in the Apple Ca^2+^-ATPase Gene Family

Analysis of codon usage bias (Figure S4) revealed a preference for guanine (G) and uracil (U) at the third codon position in the apple Ca^2+^-ATPase genes. A total of 26 preferred codons were identified: ten ending with U, eight with adenine (A), six with G, and two with cytosine (C). Most preferred codons exhibited relative synonymous codon usage (RSCU) values below 2, with the exception of AUG (methionine), which had an RSCU of 3.0 and displayed strong usage bias. This finding aligns with the conserved function of AUG as the universal eukaryotic translation initiation signal, reflecting conserved translational initiation machinery for Ca^2+^-ATPase genes. Codons GCG (alanine) and ACG (threonine) were the least utilized, both with RSCU values of 0.40.

Correlation analyses (Figure S5) demonstrated that the frequency of thymine at the third codon position (T3s) negatively correlates with the frequency of cytosine at the third codon position (C3s), the frequency of guanine at the third codon position (G3s), the Codon Adaptation Index (CAI), the Codon Bias Index (CBI), the Frequency of Optimal Codons (FOP), and the GC content at the third codon position (GC3s); the Effective Number of Codons (NC) and GC3s positively correlate with overall gene GC content; and C3s negatively correlates only with T3s and the frequency of adenine at the third codon position (A3s) but positively correlates with other indices. These results suggest that codon usage bias is influenced by base composition, gene structure, and additional factors.

### 2.7. Protein Interaction Networks and Tissue-Specific Expression of Apple Ca^2+^-ATPase Gene Family

**Figure 6:**
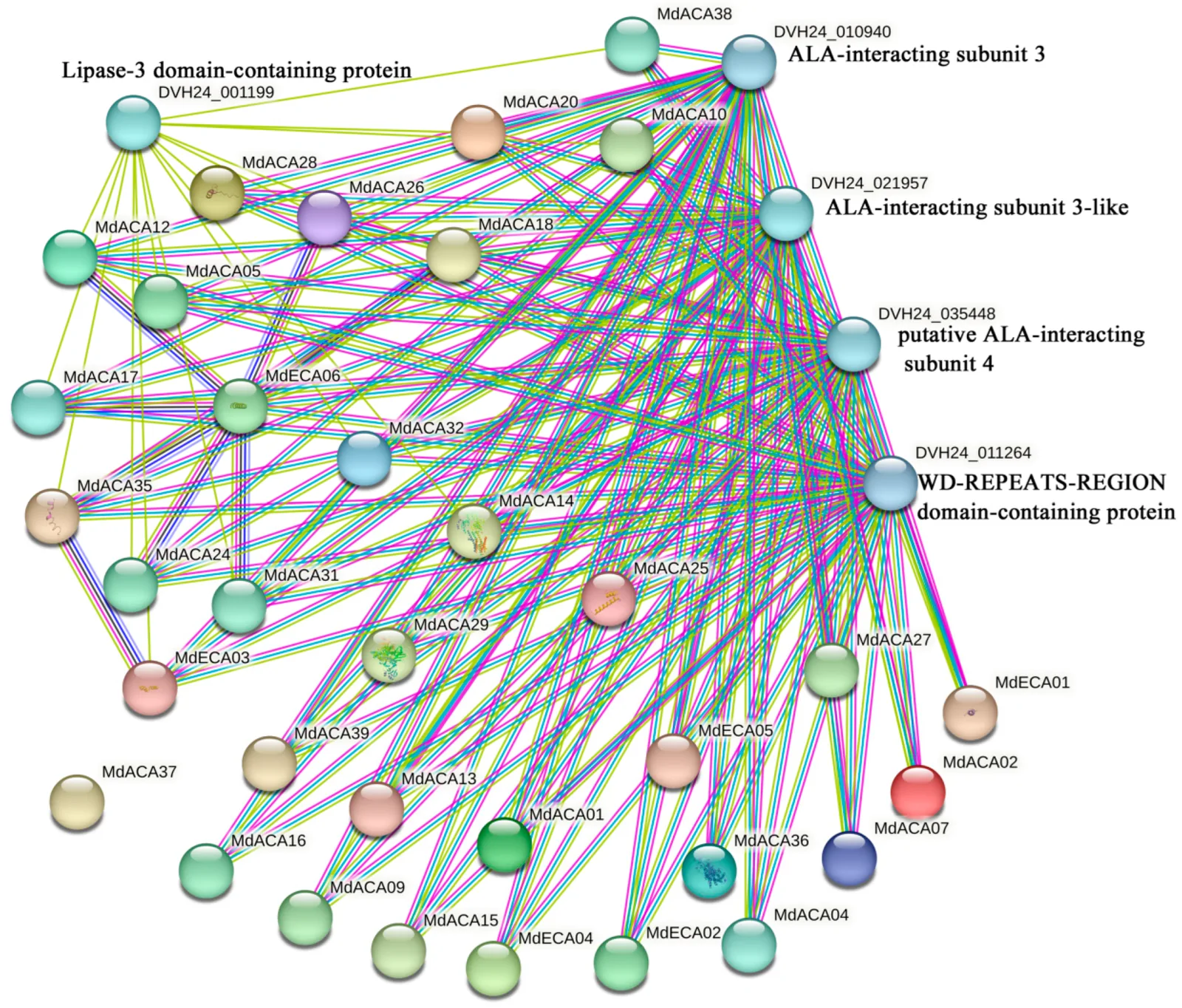
Analysis of protein–protein interactions within the apple Ca^2+^-ATPase gene family. In this network, nodes denote individual proteins, while the connecting lines indicate interactions between these proteins. The various colors of the lines correspond to distinct categories of protein interactions.

The predicted protein–protein interaction (PPI) network analysis (Figure 6) identified MdECA06 as a central hub protein, interacting with seven other proteins, including MdACA26, MdACA12, MdACA17, MdACA35, MdACA24, MdACA31, and MdACA18. Key interacting proteins, such as ALIS3 (DVH24_010940; ALA-interacting subunit 3), ALIS4 (DVH24_035448; Putative ALA-interacting subunit 4), and a WD repeat domain-containing protein (DVH24_011264), are functionally involved in plasma membrane lipid regulation, organelle function maintenance, transcriptional modification, and cell cycle control. These findings suggest that the apple Ca^2+^-ATPase family may participate in fundamental physiological processes, including cellular metabolism and membrane homeostasis, through protein interactions.

**Figure 7:**
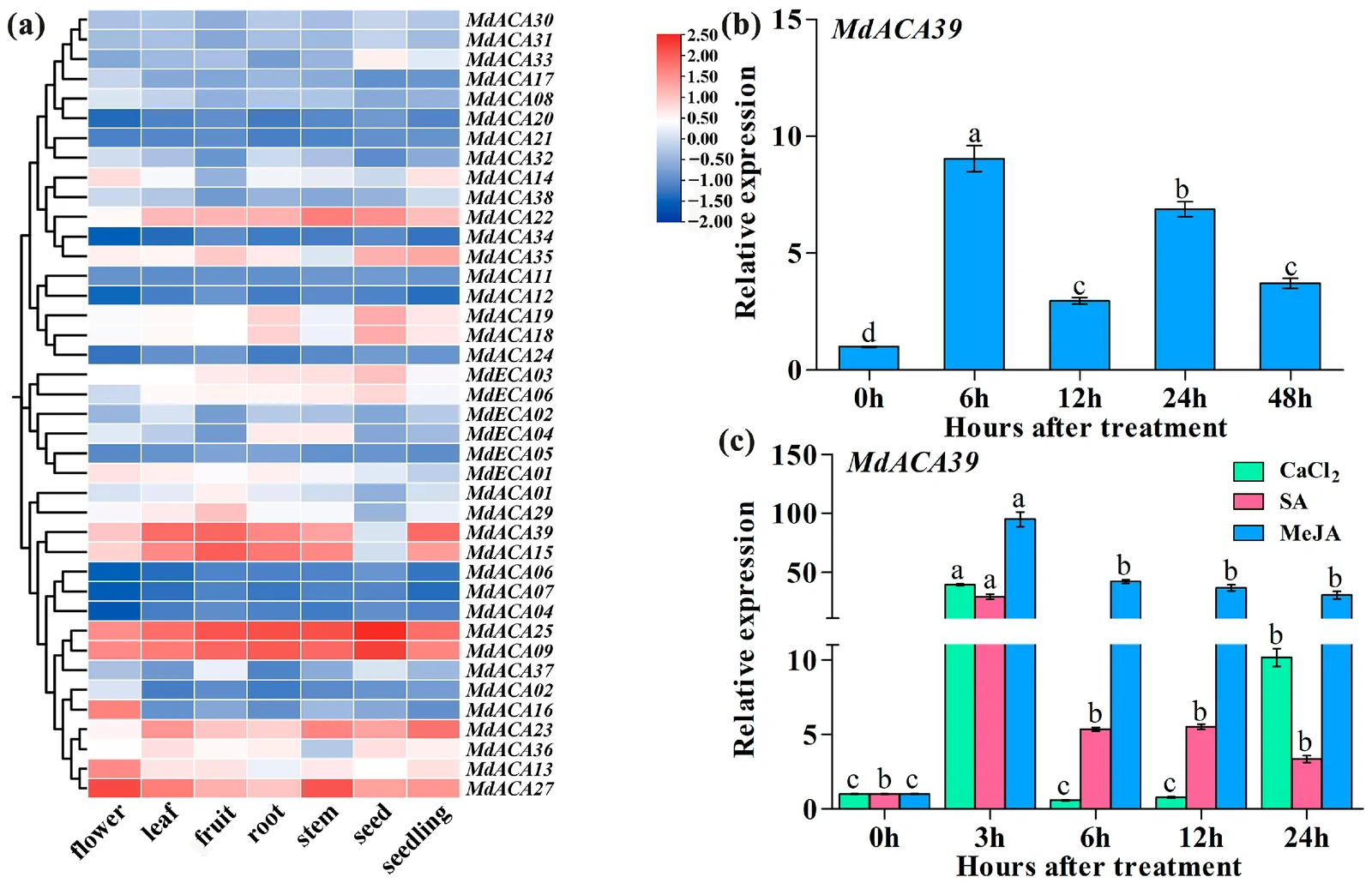
Tissue-specific expression analysis of Ca^2+^-ATPase genes and expression profiling of *MdACA39* under multiple stress treatments in apple. (**a**) Tissue-specific expression analysis of Ca^2+^-ATPase genes in apple. Red indicates high gene expression, while white indicates low gene expression. The samples correspond to the following tissues: flower (M67), fruit (M20), leaf (M14), root (GD), stem (X8877), seed (4442 × 2596), and seedling (X4102). (**b**) Analysis of the expression patterns of *MdACA39* in response to *A. alternata* infection. The y-axis represents relative gene expression levels, and the x-axis corresponds to time points at 0, 6, 12, 24, and 48 h (h). (**c**) Expression analysis of *MdACA39* under CaCl_2_, SA, and MeJA treatments. The y-axis represents relative gene expression levels, and the x-axis corresponds to the time points assessed (0, 3, 6, 12, and 24 h). *Mdtubulin* (GenBank accession number XM_008378370.4) was used as the internal reference gene. Statistical significance was determined at the 0.05 level, with differing lowercase letters indicating significant differences between time points, whereas identical lowercase letters denote the absence of statistically significant differences.

A comparative analysis utilizing the GEO database identified 40 Ca^2+^-ATPase genes with corresponding GDR locus gene identifiers, as detailed in Table S1. Tissue-specific expression profiling revealed differential expression of these 40 family genes across roots, stems, leaves, flowers, fruits, seeds, and seedlings (Figure 7a). Ten genes (*MdACA04*, *MdACA06*, *MdACA07*, *MdACA11*, *MdACA12*, *MdACA20*, *MdACA21*, *MdACA24*, *MdACA34*, and *MdECA05*) exhibited negligible expression in all tissues, whereas 30 genes displayed distinct tissue preferences. Notably, *MdACA09* and *MdACA25* were consistently highly expressed in all tissues; *MdACA15* and *MdACA39* showed low expression exclusively in seeds; *MdACA22* and *MdACA23* were weakly expressed in flowers but highly expressed elsewhere; *MdACA23* was predominantly expressed in leaves, stems, and seedlings; *MdACA13* and *MdACA16* were specifically highly expressed in floral organs. These expression patterns suggest functional differentiation among family members during apple organ development.

### 2.8. qRT-PCR Expression Analysis of Ca^2+^-ATPase Gene Family Under Multiple Stress Treatments

To explore the stress-responsive functions of the Ca^2+^-ATPase gene family, 11 *MdACA* and 2 *MdECA* genes identified in prior research were subjected to qRT-PCR analysis to characterize their expression patterns under A. *alternata* infection, CaCl_2_, SA and MeJA treatments (Figure 7b,c and Figures S6 and S7). Gene expression exhibited divergent responses to different stresses, among which *MdACA39* displayed striking upregulation under all tested conditions and represented the most responsive member. Upon *A. alternata* inoculation, *MdACA11*, *MdACA15*, *MdACA35* and *MdACA39* showed transient induction followed by repression. *MdACA39* responded fastest and strongest, peaking at 6 h with a 9.0-fold elevation. Under CaCl_2_ treatment, *MdACA39* peaked at 3 h with a 39.4-fold increase, far exceeding the induction amplitude of other induced genes. Similarly, *MdACA39* reached its maximum expression at 3 h under SA treatment (29.1-fold induction). Upon MeJA treatment, *MdACA39* showed the strongest transcriptional induction, with a 94.9-fold expression peak at 3 h. Other family members displayed milder, variable induction or repression across treatments. Collectively, these results suggest that *MdACA39* acts as a core regulator coordinating biotic stress, calcium and phytohormone signaling responses.

### 2.9. Subcellular Localization of MdACA39

**Figure 8:**
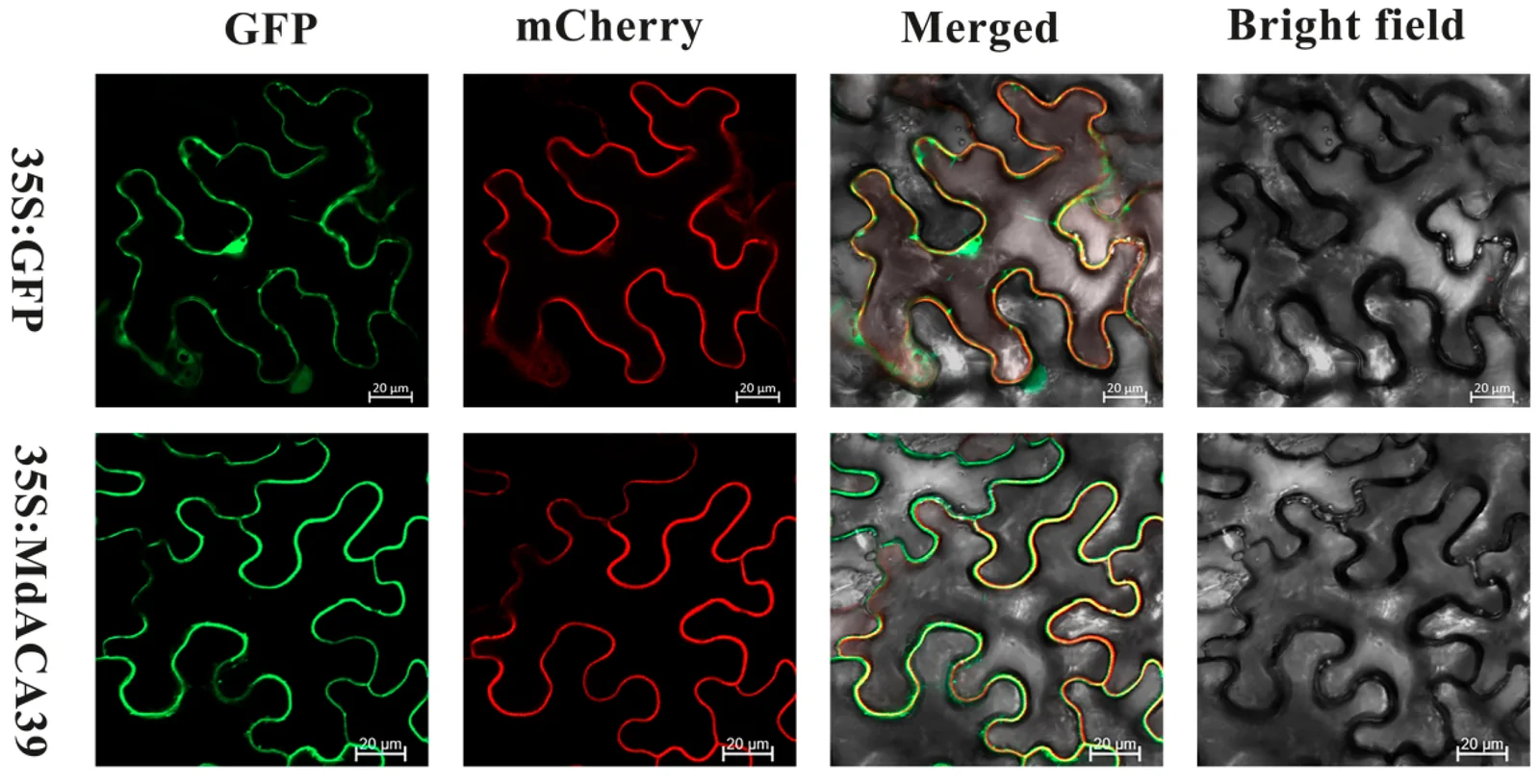
The subcellular localization of MdACA39 in *Nicotiana benthamiana* leaves. Tobacco epidermal cells were transiently transformed with either a control vector expressing GFP alone or a fusion plasmid (35S-*MdACA39*-GFP). The composite images include GFP fluorescence (**left**), mCherry fluorescence and merged images (**center**), and bright-field images (**right**). The mCherry marker (35S-*mCherry*-RFP) was used to label the membrane. Scale bar: 20 μm.

To verify the subcellular localization of MdACA39, a 35S-*MdACA39*-GFP fusion expression vector was constructed for transient transformation in *Nicotiana benthamiana* leaves, using empty 35S-GFP and 35S-*mCherry*-RFP plasma membrane marker vectors as controls (Figure 8). Confocal laser scanning microscopy revealed that GFP fluorescence from the empty vector was detected ubiquitously int the cytoplasm and nucleus of *Nicotiana benthamiana* epidermal cells, whereas the MdACA39-GFP fusion fluorescence co-localized exclusively with the plasma membrane marker. These results confirmed that MdACA39 is specifically localized to the plasma membrane, consistent with bioinformatics predictions.

### 2.10. Transient Overexpression of MdACA39 Enhances Apple Resistance to A. alternata

**Figure 9:**
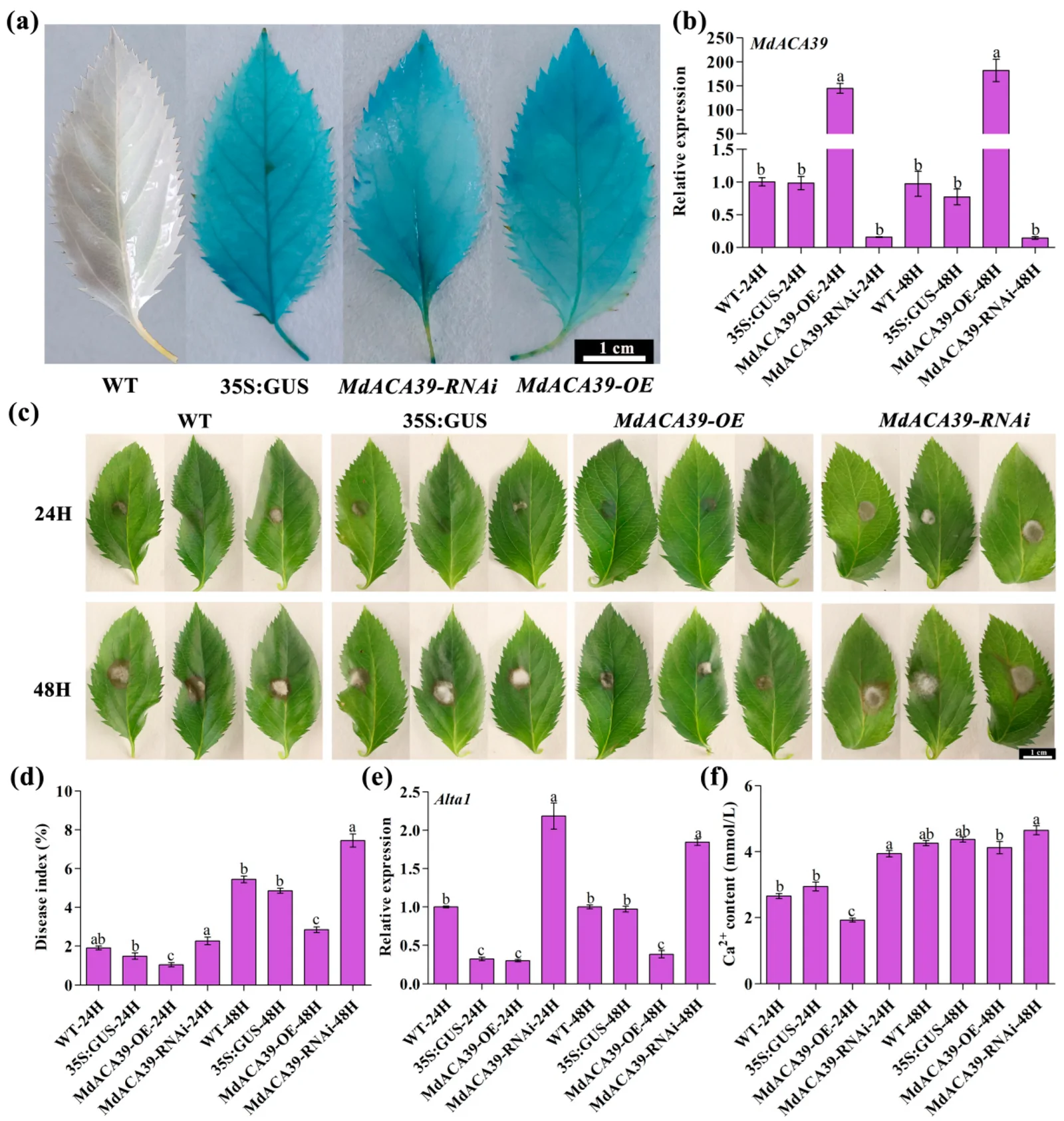
Overexpression of the *MdACA39* gene significantly enhances resistance to *A. alternata*. (**a**) presents GUS staining results for leaves from WT, 35:GUS, *MdACA39*-OE and *MdACA39*-RNAi lines, (**b**) RT-PCR analysis of *MdACA39* expression levels in leaves from these groups. (**c**) The phenotypic effects on apple leaves following inoculation with *A. alternata* in leaves from these groups. (**d**) Quantifies the disease index in leaves subjecting to *A. alternata* inoculation across the different genotypes. (**e**–**f**) qRT-PCR data for *Alta1* expression and measurements of calcium ion concentrations in leaves from these groups. WT: wild type, 35S:GUS: empty vector, *MdACA39*-OE: *MdACA39* overexpression, and *MdACA39*-RNAi: *MdACA39* RNA interference. Statistical significance was determined at the 0.05 level, with differing lowercase letters indicating significant differences between time points, whereas identical lowercase letters denote the absence of statistically significant differences.

To investigate the function of *MdACA39*, *Agrobacterium*-mediated transient expression was performed in ‘Gala-3’ apple leaves. The efficiency of the transformation system was confirmed by GUS staining and qRT-PCR analysis (Figure 9a,b). Leaves transformed with the pCAMBIA2300-35S-GUS, pCAMBIA2300-35S-*MdACA39*-GUS, and pRNAi-*MdACA39*-GUS vectors exhibited blue staining, whereas WT leaves showed no staining. qRT-PCR analysis revealed significantly elevated *MdACA39* expression in overexpressing leaves at 24 and 48 h compared to WT, empty vector, and RNAi leaves. Conversely, RNAi leaves exhibited significantly reduced expression, confirming the reliability of the system.

Following inoculation with the *A. alternata* pathogen, phenotypic observations, disease index assessments, pathogen biomass quantification via *Alta1* gene expression [28], and calcium ion content measurements were conducted at 24 and 48 h (Figure 9c–f). The results demonstrated that overexpression of *MdACA39* significantly reduced leaf lesion area and disease severity, whereas RNAi-mediated silencing of *MdACA39* accelerated lesion expansion and exacerbated disease symptoms at 48 h. Pathogen biomass analysis revealed that *Alta1* expression was significantly lower in *MdACA39*-OE leaves compared to WT and empty vector controls (except at 24 h) and significantly higher in *MdACA39*-RNAi leaves. Calcium ion content was significantly decreased in *MdACA39*-OE leaves and elevated in *MdACA39*-RNAi leaves at 24 h; at 48 h, *MdACA39*-RNAi leaves maintained higher calcium levels than *MdACA39*-OE leaves but were comparable to WT and empty vector controls. These results indicate that *MdACA39* may participate in calcium homeostasis regulation and be associated with the activation of immune signaling to enhance resistance to *A. alternata*.

### 2.11. Expression Analysis of Disease Resistance-Related Genes Mediated by MdACA39

**Figure 10:**
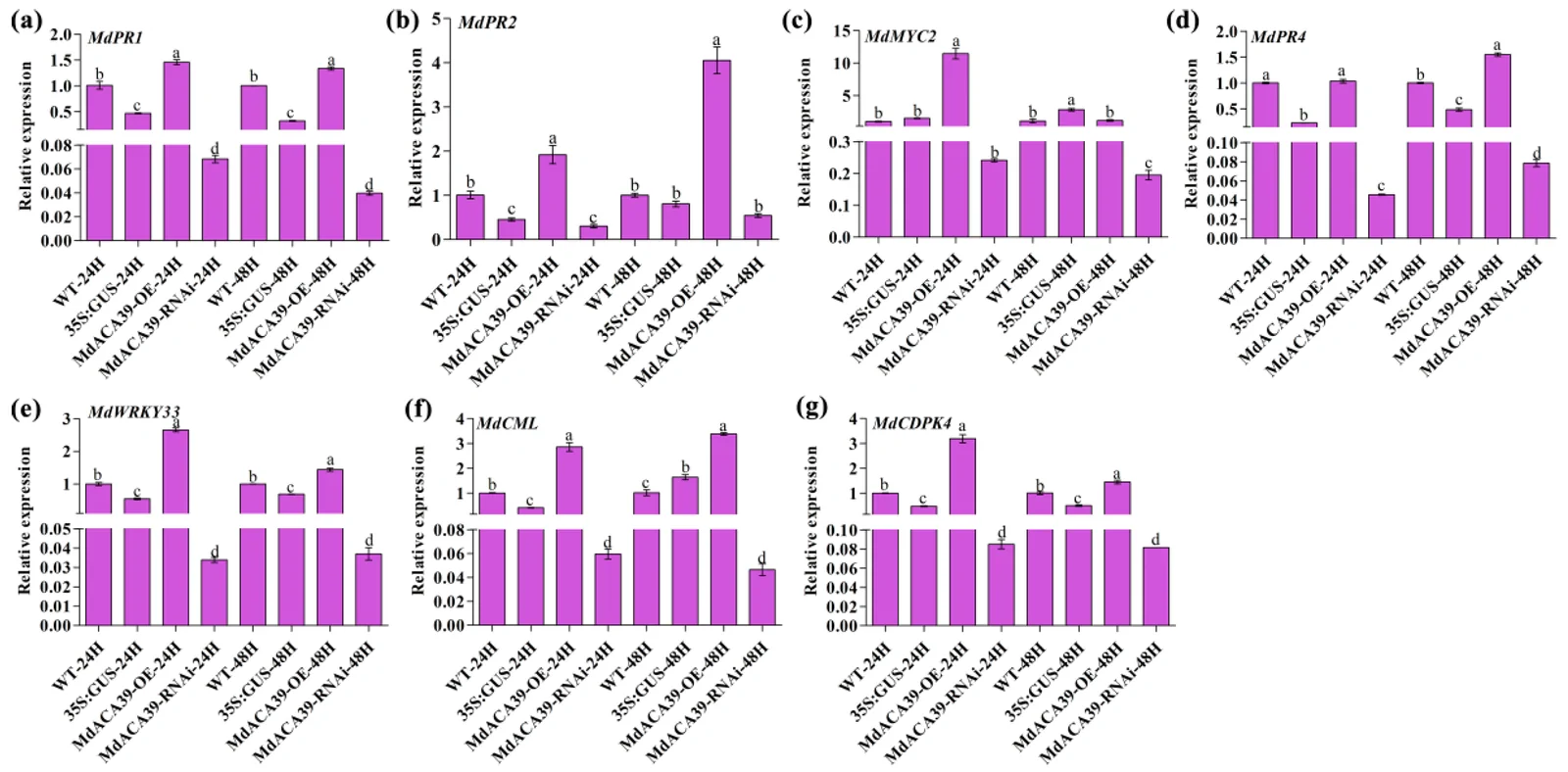
Effects of *A. alternata* inoculation on transcript profiles of disease resistance marker genes in *MdACA39*-OE apple leaves. (**a**–**g**) The relative expression abundance of *MdPR1* (**a**), *MdPR2* (**b**), *MdMYC2* (**c**), *MdPR4* (**d**), *MdWRKY33* (**e**), *MdCML* (**f**), and *MdCDPK4* (**g**) isolated from *MdACA39*-OE leaf tissues. WT: wild type, 35S:GUS: empty vector, *MdACA39*-OE: *MdACA39* overexpression, and *MdACA39*-RNAi: *MdACA39* RNA interference. Statistical significance was determined at the 0.05 level, with differing lowercase letters indicating significant differences between time points, whereas identical lowercase letters denote the absence of statistically significant differences.

To elucidate the molecular mechanisms underlying *MdACA39*-mediated disease resistance, the expression of SA pathway genes (*MdPR1* and *MdPR2*), JA pathway genes (*MdMYC2* and *MdPR4*), calcium signaling components (*MdCML* and *MdCDPK4*), and the disease resistance transcription factor *MdWRKY33* was analyzed by qRT-PCR at 24 and 48 h [29] (Figure 10a–g). At 24 h, *MdACA39*-OE leaves exhibited significantly elevated expression of *MdPR1*, *MdPR2*, *MdMYC2*, *MdWRKY33*, *MdCML*, and *MdCDPK4* relative to WT and empty vector controls. Conversely, *MdACA39*-RNAi leaves showed significantly reduced expression of *MdPR1*, *MdPR4*, *MdWRKY33*, *MdCML*, and *MdCDPK4*. At 48 h, *MdACA39*-OE leaves maintained higher expression of *MdPR1*, *MdPR2*, *MdPR4*, *MdWRKY33*, *MdCML*, and *MdCDPK4*, whereas *MdACA39*-RNAi leaves exhibited lower expression of *MdPR1*, *MdMYC2*, *MdPR4*, *MdWRKY33*, *MdCML*, and *MdCDPK4* compared to WT and empty vector controls. These results indicate that *MdACA39* may positively regulate multiple disease resistance signaling pathways, thereby promoting resistance gene expression and enhancing apple defense against *A. alternata*.

### 2.12. Analysis of the Antioxidant System in MdACA39-Transformed Apple Leaves

**Figure 11:**
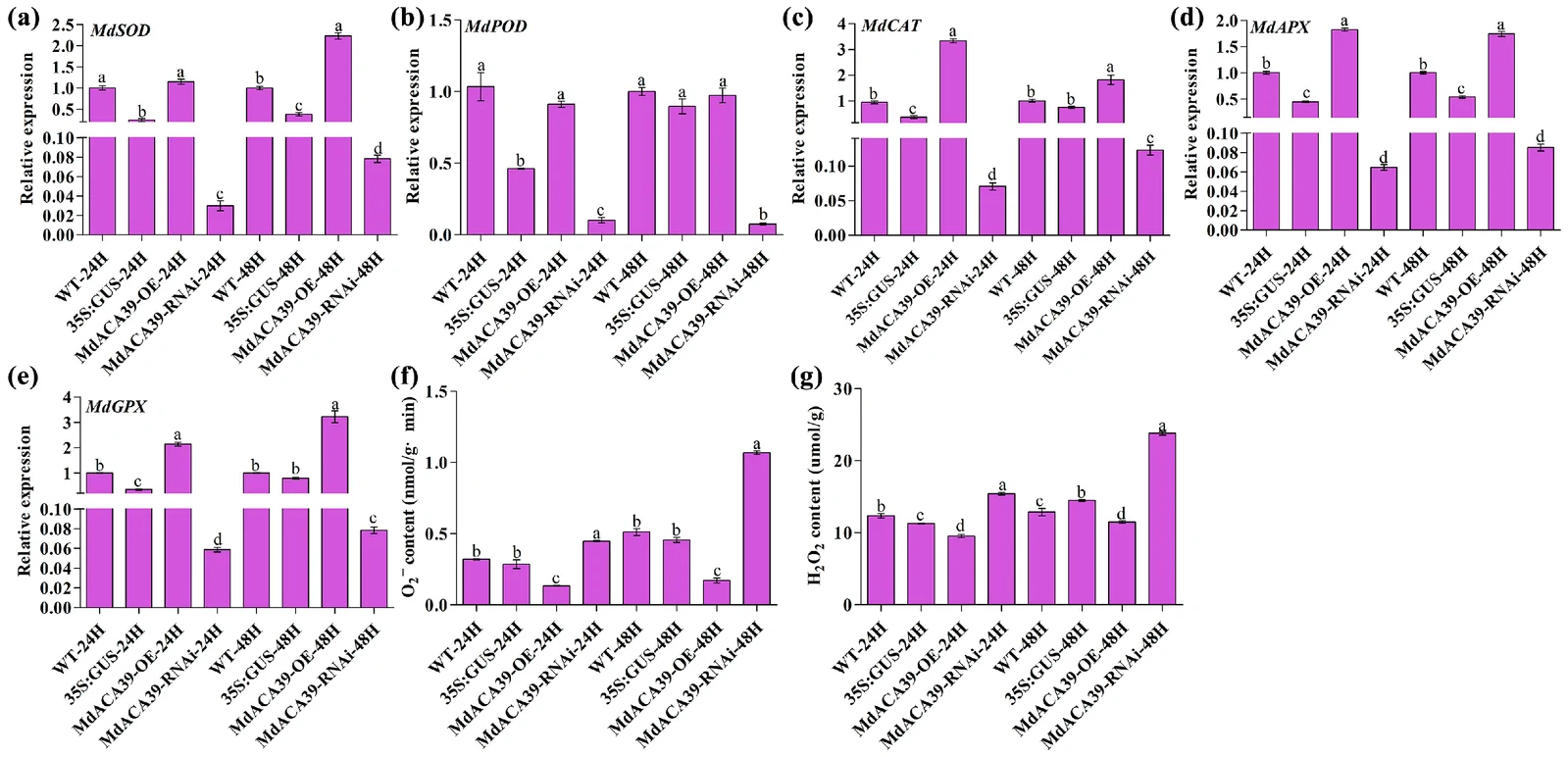
The Analysis of the expression levels of antioxidant enzyme genes and the quantification of O_2_^−^ and H_2_O_2_ concentrations in *MdACA39*-OE leaves. (**a**–**e**) The expression profiles of *MdSOD* (**a**), *MdPOD* (**b**), *MdCAT* (**c**), *MdGPX* (**d**), and *MdAPX* (**e**) genes in *MdACA39*-OE leaves. (**f**,**g**) The measured levels of O_2_^−^ and H_2_O_2_, respectively, in these genetically modified leaves. WT: wild type, 35S:GUS: empty vector, *MdACA39*-OE: *MdACA39* overexpression, and *MdACA39*-RNAi: *MdACA39* RNA interference. Statistical significance was determined at the 0.05 level, with differing lowercase letters indicating significant differences between time points, whereas identical lowercase letters denote the absence of statistically significant differences.

To further elucidate the disease resistance mechanism conferred by *MdACA39*, the expression of antioxidant enzyme-related genes and the levels of reactive oxygen species (ROS), specifically superoxide anion (O_2_^−^) and hydrogen peroxide (H_2_O_2_), were evaluated using qRT-PCR and biochemical assays at 24 and 48 h (Figure 11a–g). The qRT-PCR results showed that the antioxidant genes *MdSOD*, *MdCAT*, *MdGPX*, and *MdAPK* were significantly upregulated in *MdACA39*-OE leaves (except *MdPOD*). Correspondingly, the accumulation of O_2_^−^ and H_2_O_2_ was markedly reduced in the *MdACA39*-OE leaves. Conversely, *MdACA39*-RNAi-mediated silencing of *MdACA39* inhibited the expression of antioxidant enzyme genes and caused excessive ROS accumulation in apple leaves. These results suggest that *MdACA39* activates the antioxidant defense system to scavenge pathogen-induced ROS, mitigate oxidative damage, and ultimately enhance apple resistance to *A. alternata*.

## 3. Discussion

### 3.1. Evolutionary and Structural Basis Underlying the Functional Conservation and Divergence of Apple Ca^2+^-ATPase Gene Family

Ca^2+^-ATPases constitute an essential subfamily of the plant P-type ATPase superfamily, playing a central role in maintaining cytosolic Ca^2+^ homeostasis in planta. These enzymes are extensively involved in multiple fundamental biological processes, including plant growth and development, calcium-mediated signal transduction, responses to abiotic stresses, and immune defense mechanisms [16,18,25,30,31]. In this study, a total of 45 Ca^2+^-ATPase genes were identified in the *Malus domestica* genome. These genes are unevenly distributed across 14 apple chromosomes, with only one gene anchored to Chr00. This gene family comprises 39 *MdACA* and 6 *MdECA* genes (Figure 1), a number substantially greater than that reported in *Arabidopsis thaliana* (11 *ACAs* and 4 *ECAs*) and *Oryza sativa* (12 *ACAs* and 3 *ECAs*), while comparable to that of *Triticum aestivum* (33 *ACAs* and 9 *ECAs*) [12,22]. These results indicate that the Ca^2+^-ATPase gene family has undergone remarkable expansion during the evolutionary history of apple.

Phylogenetic analyses categorized the Ca^2+^-ATPase genes from apple, *Arabidopsis*, and rice into five distinct subfamilies: Groups A, B, C, and D correspond to the ACA class, whereas Group E aligns with the ECA class. Notably, group D represents an apple-specific ACA subfamily (Figure 2), highlighting both the high conservation and species-specific functional divergence within this gene family. This evolutionary pattern is consistent with previous reports in soybean, banana, and sugar beet [14,15,32]. Gene duplication events are recognized as pivotal drivers of gene family expansion and functional diversification in plants [33]. Unlike *Arabidopsis*, which exhibits no gene duplication within its Ca^2+^-ATPase gene family, most soybean Ca^2+^-ATPase genes have undergone duplication [12,14]. Collinearity analysis identified 25 pairs of intra-species collinear genes and 135 pairs of inter-species homologous genes within the apple Ca^2+^-ATPase family (Figure 3 and Figure 4), with segmental duplication emerging as the predominant mechanism underlying family expansion. The number of homologous genes shared between apple and dicotyledonous species such as *Arabidopsis* and grape is markedly higher than that shared with monocotyledons like rice and maize, consistent with the established phylogenetic relationships among these plant species.

Following duplication, gene pairs may undergo neofunctionalization, subfunctionalization, or nonfunctionalization [34]. All duplicated gene pairs exhibited Ka/Ks ratios below 1 (Figure S1), indicative of strong purifying selection and limited sequence divergence after duplication. This evolutionary constraint further supports the high evolutionary conservation of the apple Ca^2+^-ATPase gene family, consistent with the phylogenetic clustering results [35]. In plants, Ca^2+^-ATPases predominantly localize to the plasma membrane and various internal membrane systems. In *Arabidopsis*, AtACA8, AtACA9, and AtACA10 are localized to the plasma membrane [36,37,38]; AtACA4 and AtACA11 are associated with the vacuolar membrane [39,40]; and AtECA3 is localized to the ER and Golgi apparatus [41,42]. Subcellular localization predictions for apple Ca^2+^-ATPase proteins indicate predominant localization to the plasma membrane, ER, and vacuole (Table S2), aligning with localization characteristics reported in Arabidopsis, banana, and lettuce [14,16,36]. Subcellular localization critically influences protein function; thus, the plasma membrane localization of apple Ca^2+^-ATPases likely facilitates the regulation of intracellular Ca^2+^ homeostasis through extrusion of Ca^2+^ ions or their sequestration within organelles such as the ER [30].

Variations in exon and intron numbers within gene structures play a key role in the functional evolution of genes [43]. Structural and conserved motif analyses revealed that core motifs within apple Ca^2+^-ATPase proteins are highly conserved, ensuring their fundamental calcium transport function. Meanwhile, variations in exon number and untranslated region (UTR) sequences provide a structural basis for functional diversification within the family (Figure 5a,c). This structural divergence pattern is consistent with previous studies on Ca^2+^-ATPase families in *Solanaceous* species and *alfalfa* [14,44,45].

### 3.2. Regulatory Characteristics and Expression Patterns of Apple Ca^2+^-ATPase Genes in Growth Development and Stress Response

Plant transcription factors regulate gene expression by binding to *cis*-acting elements within promoter regions, thereby orchestrating plant growth, development, and stress responses [46]. Promoter analysis (Figure 5b) of apple Ca^2+^-ATPase genes revealed widespread enrichment of *cis*-elements responsive to light, hormones, and various stresses, suggesting that this gene family integrates diverse environmental and hormonal signals to regulate apple growth, development, and stress adaptation. This dual characteristic of functional conservation and diversity aligns with findings reported in other plant species [12,14]. Protein–protein interaction analyses demonstrated that MdECA06 interacts with MdACA and MdECA proteins, as well as with ALIS3 and ALIS4 proteins (Figure 6), implicating these interactions in calcium signaling regulation and expanding the molecular regulatory network of Ca^2+^-ATPases. Nevertheless, further experimental validation is required to confirm these interactions [47]. This interaction network suggests that apple Ca^2+^-ATPases may coordinate with lipid transporters to maintain membrane integrity and function under stress conditions.

In species such as *Arabidopsis* and wheat, Ca^2+^-ATPase genes exhibit pronounced tissue-specific expression patterns. For instance, *AtACA2* transcripts are abundant in pollen, roots, and leaves [12,18,22]. Consistent with these findings, tissue-specific expression profiling in apple revealed significant expression preferences among Ca^2+^-ATPase family members (Figure 7a): *MdACA09* and *MdACA25* are highly expressed across multiple tissues; *MdACA15* and *MdACA39* show low expression in seeds; *MdACA22* and *MdACA23* exhibit weak expression in flowers; *MdACA23* is predominantly expressed in leaves, stems, and seedlings; and *MdACA13* and *MdACA16* display elevated expression specifically in floral organs. These differential expression patterns suggest distinct functional roles for family members in regulating vegetative and reproductive organ development. Furthermore, *AtACA12* and *AtACA13* are significantly induced upon pathogen infection [20]. Similarly, most Ca^2+^-ATPase genes in wheat are transcriptionally activated under *Fusarium graminearum* infection [22], and banana Ca^2+^-ATPase genes show elevated expression in response to Ca^2+^ treatment [14]. Expression analyses under various stress conditions demonstrated that most Ca^2+^-ATPase family members respond to *A. alternata* infection, calcium ion treatment, SA, and MeJA stimuli in apple. Notably, *MdACA39* exhibited a markedly stronger response than other genes (Figure 7b,c), suggesting its potential role as a central stress-responsive gene within the family.

### 3.3. Immune Regulatory Function and Mechanistic Insight of MdACA39 in Apple Disease Resistance

Previous research has established that ACA proteins within the plant Ca^2+^-ATPase family perform dual functions in immune regulation. They act as positive regulators of pathogen-associated molecular pattern (PAMP)-triggered immunity (PTI) by modulating specific calcium signals [24], while also serving as negative regulators to mitigate excessive immune responses and prevent self-inflicted damage [21,27,48]. In this study, MdACA39 was localized to the plasma membrane and demonstrated a positive regulatory role in apple resistance to *A. alternata* (Figure 8 and Figure 9). Transient overexpression of *MdACA39* activated SA and JA defense pathways, calcium signaling cascades, and antioxidant systems, resulting in reduced ROS accumulation and decreased pathogen biomass (Figure 10 and Figure 11). Conversely, RNAi-mediated silencing of *MdACA39* significantly diminished leaf disease resistance.

The immune-regulatory function of *MdACA39* is consistent with the positive immune roles of plasma membrane-localized *AtACA8* and *AtACA10* in *Arabidopsis thaliana* yet differs from the negative immune regulation mediated by tonoplast-localized *AtACA4/AtACA11* and ER-localized *OsACA5*. Such functional divergence highlights that subcellular localization acts as a key determinant governing the immune functions of Ca^2+^-ATPases across plant species [21,24,48]. As a plasma membrane-resident Ca^2+^ pump, *MdACA39* probably orchestrates Ca^2+^ shuttling across the plasma membrane to shape dynamic intracellular Ca^2+^ signatures. The Ca^2+^ signal may subsequently be sensed by calmodulin (CaM), further triggering downstream MAPK cascades and ROS signaling to activate defense-related genes in apple. In addition, *MdACA39* may be mediated Ca^2+^ homeostasis facilitates ROS scavenging, which may alleviate excessive immune activation and mitigate growth suppression, thereby fine-tuning the growth–defense trade-off.

Collectively, this study preliminarily reveals the regulatory mechanism of *MdACA39* in apple disease resistance. The transient overexpression and RNAi assays conducted on apple leaves offer supportive evidence for its immune function, yet this experimental system has inherent drawbacks. Transient transformation only enables short-duration gene expression and suffers from genetic instability, which restricts the reliability and generalizability of the obtained phenotypes. Therefore, stable genetic transformation, gene knockout approaches, and protein interaction analyses are required to thoroughly characterize the *MdACA39* may be dependent Ca^2+^ signaling cascade governing apple disease resistance. Further research along these lines will supply elite gene resources and establish a theoretical framework for molecular breeding to enhance fungal disease resistance in apple.

## 4. Materials and Methods

### 4.1. Experimental Materials and Treatment Procedures

The present study was conducted in the Fruit Tree Physiology and Biotechnology Laboratory at the College of Horticulture, Gansu Agricultural University, located in Lanzhou City, Gansu Province. The experimental materials consisted of in vitro-cultured apple seedlings of the ‘Gala-3’ cultivar, propagated via stem segment subculture. The subculture medium comprised basal Murashige and Skoog (MS) medium supplemented with 30 g·L^−1^ sucrose, 6.0 g·L^−1^ agar, 0.3 mg·L^−1^ 6-benzylaminopurine (6-BA), 0.2 mg·L^−1^ indole-3-acetic acid (IAA), and 0.1 mg·L^−1^ gibberellic acid (GA_3_). Artificial climate conditions followed the protocol described [49]. After 35 days of subculture, healthy, uniformly growing, and contamination-free seedlings were selected for rooting culture, which used half-strength MS medium supplemented with 30 g·L^−1^ sucrose, 6.0 g·L^−1^ agar, 0.4 mg·L^−1^ indole-3-butyric acid (IBA), and 1 mg·L^−1^ IAA. Following an additional 35 days of continuous culture, seedlings underwent subsequent stress treatments and sampling. Functional leaves from healthy tissue-cultured seedlings were excised at the petiole base and placed on MS solid medium. *A. alternata* was isolated from symptomatic apple leaves collected from apple orchards on the Loess Plateau, Gansu Province, China, and its pathogenicity was verified. Inoculation with the *A. alternata* was performed according to the methodology [50]. Leaf samples were collected at 0, 6, 12, 24, and 48 h. For abiotic stress and hormonal treatments, the MS medium was supplemented with 150 mmol·L^−1^ calcium chloride dihydrate (CaCl_2_·2H_2_O), 0.1 mmol·L^−1^ SA, or 0.1 mmol·L^−1^ MeJA, respectively. Samples were harvested at 0, 3, 6, 12, and 24 h following treatment. All collected samples were immediately wrapped in aluminum foil, flash-frozen in liquid nitrogen, and stored at −80 °C for subsequent molecular and physiological analyses.

### 4.2. Identification of Apple Ca^2+^-ATPase Gene Family Members and Analysis of Their Physicochemical Properties

The gene IDs of *Arabidopsis thaliana* Ca^2+^-ATPase family members were obtained from a previously published study [12]. Corresponding full-length sequences, coding sequences (CDS), amino acid sequences, and complementary DNA (cDNA) sequences were retrieved from the TAIR database (https://www.arabidopsis.org/; accessed on 16 July 2024). Apple GDDH13 v1.1 reference genome sequences and annotations were downloaded from the Phytozome13 database (https://phytozome-next.jgi.doe.gov/; accessed on 16 July 2024). Homologous sequence alignment was performed using TBtools-II v2.105 software to identify putative apple Ca^2+^-ATPase homologous genes, and the corresponding protein, CDS, and full-length gene sequences were extracted. The Hidden Markov Model (HMM) profile PF00690 (Cation_ATPase_N), characteristic of the Ca^2+^-ATPase family, was used as a reference for gene family screening [14,15]. Conserved domain verification was conducted sequentially using the SMART database (http://smart.embl.de/; accessed on 31 July 2024), the NCBI Conserved Domain Database (CDD) (https://www.ncbi.nlm.nih.gov/cdd/; accessed on 31 July 2024), and the Pfam database (http://pfam.xfam.org/), culminating in the definitive identification of apple Ca^2+^-ATPase family members. The physicochemical properties of the identified proteins, including molecular weight, theoretical isoelectric point, and hydrophobicity, were analyzed using the ExPASy ProtParam tool (https://web.expasy.org/protparam/; accessed on 31 July 2024) [51].

### 4.3. Chromosomal Localization, Subcellular Localization Prediction, and Secondary Structure Analysis of the Apple Ca^2+^-ATPase Gene Family

The chromosomal localization of Ca^2+^-ATPase genes was determined by analyzing the apple genome annotation file using TBtools-II v2.105 software [52], followed by graphical visualization. Subcellular localization predictions for the protein family were performed using the WoLF PSORT online tool (https://www.genscript.com/wolf-psort.html; accessed on 1 August 2024). The secondary structural composition, including the proportions of α-helix, β-turn, random coil, and extended strand elements, was analyzed using the PRABI SOPMA tool (https://npsa-prabi.ibcp.fr/cgi-bin/npsa_automat.pl?page=npsa_sopma.html; accessed on 1 August 2024).

### 4.4. Phylogenetic Tree Construction and Collinearity Analysis of the Apple Ca^2+^-ATPase Gene Family

Protein sequences of Ca^2+^-ATPase from apple, *Arabidopsis*, and rice were retrieved from the Phytozome v13 database. Multiple sequence alignments were performed using ClustalW within MEGA version 11.0 software, and a phylogenetic tree was constructed using the Neighbor-Joining method with the *p*-distance model for amino acid sequences and 1000 bootstrap replicates [53]. Genome annotation files for apple, *Arabidopsis*, grape, rice, and maize were imported into TBtools-II v2.105 software for cross-species collinearity analysis and visualization using the Multiple Synteny Plot tool. Intraspecific collinearity within apple was analyzed with MCScanX and visualized using the Advanced Circos tool. Synonymous (Ks) and nonsynonymous (Ka) substitution rates, along with Ka/Ks ratios for duplicated gene pairs, were calculated to assess the evolutionary selection pressures acting on this gene family.

### 4.5. Analysis of Conserved Motifs, Cis-Acting Regulatory Elements, and Gene Structure in the Apple Ca^2+^-ATPase Gene Family

The conserved motifs of apple Ca^2+^-ATPase proteins were identified using the MEME suite (https://meme-suite.org/meme/tools/meme; accessed on 2 August 2024), with the maximum number of predicted conserved motifs set to ten. The structural features of Ca^2+^-ATPase genes, including exons, introns, and untranslated regions (UTRs), were extracted from the apple genome GFF3 annotation file using TBtools-II v2.105. Promoter regions, defined as 2000 base pairs upstream of the ATG start codon, were retrieved and analyzed for cis-acting regulatory elements via the PlantCARE database (http://bioinformatics.psb.ugent.be/webtools/plantcare/html/; accessed on 3 August 2024) [54]. The types, quantities, and potential functions of *cis*-elements were statistically summarized. Finally, TBtools-II v2.105 was used to integrate and visualize the conserved motifs, gene structures, and cis-acting element distributions of the apple Ca^2+^-ATPase gene family.

### 4.6. Codon Usage Bias and Protein Interaction Network Analysis of the Apple Ca^2+^-ATPase Gene Family

The coding sequences (CDS) of apple Ca^2+^-ATPase genes were analyzed to assess codon usage bias. Relative synonymous codon usage (RSCU) values for each gene were calculated using CodonW v1.4.2 software. The data were organized in Microsoft Excel, and correlation analysis along with visual mapping of RSCU distribution were performed using Origin 2021 and RStudio R-4.6.1 software. Protein sequences of the Ca^2+^-ATPase gene family were submitted to the STRING database (https://cn.string-db.org/; accessed on 5 August 2024) to construct a protein–protein interaction (PPI) network, facilitating the prediction of core interacting proteins and regulatory pathways.

### 4.7. Tissue-Specific Expression Analysis of the Apple Ca^2+^-ATPase Gene Family

The apple multi-tissue transcriptome dataset (GSE42873), encompassing root, stem, leaf, flower, fruit, seed, and seedling tissues, was obtained from the NCBI Gene Expression Omnibus (GEO) database. The gene ID correspondence for apple Ca^2+^-ATPase gene family members was confirmed using the Rosaceae Genome Database (GDR) (https://www.rosaceae.org/; accessed on 5 August 2024). The processed data were compiled in Excel, and heatmap visualization and hierarchical clustering of gene expression profiles were performed using TBtools-II v2.105 to illustrate tissue-specific expression patterns. The pairwise similarity of expression patterns was calculated using the Pearson correlation coefficient, and average linkage was used for cluster merging.

### 4.8. Cloning of MdACA39 and Construction of MdACA39-OE and MdACA39-RNAi Vectors

The full-length coding sequence (CDS) of *MdACA39* was obtained from Phytozome 13, and specific cloning primers were designed (Table S4). Total RNA was isolated from young leaves of ‘Gala-3’ apple seedlings and reverse-transcribed to cDNA for amplifying the *MdACA39* CDS via PCR. Purified PCR fragments were inserted into the pMD19-T vector and transformed into *E*. *coli* DH5α competent cells. Using sequence-confirmed plasmids as templates, *MdACA39* was reamplified with primers carrying *Sal*I and *Stu*I restriction sites (Table S4). The purified fragment was double-digested and ligated into the pCAMBIA2300-GUS vector to generate the 35S-*MdACA39*-GUS OE construct, which was validated by restriction enzyme digestion and sequencing. For RNAi vector construction, a synthetic PDK intron sequence was inserted into the pCAMBIA2300-GUS vector using primers with *Sal*I and *Stu*I sites. A 288 bp gene-specific fragment of the *MdACA39* gene was identified, and primers were designed accordingly. The sense primer incorporated recognition sites for the restriction enzymes *Pst*I and *Sal*I, while the antisense primer included sites for *Stu*I and *Xba*I (Table S4). These amplified fragments were then ligated flanking the PDK intron sequence to construct the pRNAi-*MdACA39*-GUS vector.

### 4.9. Subcellular Localization Analysis of MdACA39

Specific primers containing *Bgl*II and *Spe*I restriction sites were designed to amplify the *MdACA39* CDS without the stop codon (Table S4). This fragment was subsequently cloned into the pCAMBIA1302-GFP vector to generate the 35S-*MdACA39*-GFP fusion expression construct. After sequence verification, the empty pCAMBIA1302 vector, the fusion construct, and the plasma membrane marker vector (35S-*mCherry*-RFP) were separately transformed into *Agrobacterium tumefaciens* strain GV3101 via heat shock. *Agrobacterium* cultures harboring the target and marker vectors were mixed in equal proportions (OD_600_ = 0.5) and co-infiltrated into the leaves of five-week-old *Nicotiana benthamiana* plants through transient transformation. Following infiltration, the tobacco plants were incubated in darkness for 24 h and then transferred to a growth chamber under a 16 h light/8 h dark photoperiod for 48 h. Subcellular localization of MdACA39-GFP was observed and imaged using a Zeiss LSM 800 laser scanning confocal microscope (Carl Zeiss Microscopy, Oberkochen, Germany).

### 4.10. Functional Verification of the MdACA39 Gene and Measurement of Physiological Parameters

The pCAMBIA2300-GUS, pCAMBIA2300-*MdACA39*-GUS, and pRNAi-*MdACA39*-GUS vectors were individually transformed into *Agrobacterium tumefaciens* strain GV3101. Positive *Agrobacterium* strains were cultured to an OD_600_ of 0.6 and then diluted to 0.5 for vacuum infiltration-mediated transient transformation of ‘Gala-3’ apple leaves [55]. Following transformation, GUS staining and qRT-PCR were performed to assess *MdACA39* expression levels and validate the transient expression system. Uniformly grown WT, empty vector, *MdACA39*-OE, and *MdACA39*-RNAi apple leaves were inoculated with *A. alternata*. Samples were photographed and collected at 24 and 48 h. Disease lesion areas were quantified using ImageJ2 software to evaluate disease severity [56]. The experiment included three biological replicates, each consisting of at least 15 leaves. GUS staining was performed following the protocol described [57]. Leaf calcium ion content was measured using the GENMED Plant Calcium Ion Concentration Detection Kit (GMS50097.6 v.A, GENMED, Boston, MA, USA). O_2_^−^ and H_2_O_2_ levels were quantified using assay kits from Lyle Biotechnology Company (LE-1-162 and LE-1-164, Hefei, China).

### 4.11. RNA Extraction and qRT-PCR Analysis

Total RNA was extracted from apple leaves using the Omega Plant RNA Extraction Kit (R6827-01, Omega Bio-tek, Norcross, GA, USA). RNA integrity was assessed by 1% agarose gel electrophoresis, and concentration and purity were measured using microspectrophotometry. First-strand cDNA was synthesized by reverse transcription using 1 μg of qualified total RNA with the Aikery Evo-M-MLV Reverse Transcription Kit (AG11728, Accurate Biology, Changsha, Hunan, China). Gene-specific qRT-PCR primers were designed based on the coding sequences (CDS) of target genes retrieved from Phytozome 13 and verified using the NCBI Primer 3 BLAST tool (Table S5). Quantitative real-time PCR (qRT-PCR) was performed on an ABI 7500 real-time PCR system using the Aikery SYBR Green Pro Taq HS qPCR Kit (AG11701). The 20 μL reaction mixture contained 1 μL cDNA template, 1 μL forward primer, 1 μL reverse primer, 10 μL SYBR Master Mix, and 7 μL nuclease-free water. *Mdtubulin* (GenBank accession number XM_008378370.4) was used as the normalization gene. Relative gene expression levels were calculated using the 2^−ΔΔCt^ method [58].

### 4.12. Statistical Analysis

All experimental data were obtained from three independent biological replicates, each comprising three technical replicates, and are presented as mean ± standard error (SE). Raw data were organized using Microsoft Excel 2010. One-way analysis of variance (ANOVA) was performed using SPSS version 26.0; the Shapiro–Wilk test and Levene’s test were performed to verify normality of distribution and homogeneity of variance before analysis. Repeated measures ANOVA was used to analyze data measured at sequential time points. Mauchly’s test was applied to assess sphericity, and Greenhouse–Geisser correction was applied when the sphericity assumption was violated. Tukey’s test was used for multiple comparisons. A *p* < 0.05 was considered statistically significant. All statistical graphs were generated using GraphPad Prism version 8.0.

## 5. Conclusions

This study performed a systematic identification and bioinformatic analysis of the Ca^2+^-ATPase gene family in *Malus domestica*. In total, 45 family members were identified and classified into two subfamilies (MdACA and MdECA), which are unevenly distributed on 14 apple chromosomes, with one gene located on unanchored scaffolds (Chr00). Phylogenetic analysis divided these genes into five conserved subgroups, among which Group D is apple-specific. Segmental duplication dominated gene family expansion, and genes underwent strong purifying selection. Expression screening revealed that *MdACA39* was significantly induced by *A. alternata* infection, Ca^2+^, SA and MeJA treatments. Subcellular localization confirmed that MdACA39 resides on the plasma membrane. Transient expression assays further verified that *MdACA39* positively enhances apple resistance to *A. alternata* by triggering defense gene expression, boosting antioxidant capacity and sustaining calcium homeostasis. Our results elucidate the evolutionary characteristics of apple Ca^2+^-ATPases and reveal the positive disease-resistance function of *MdACA39*, providing theoretical support and gene resources for breeding apple varieties with improved fungal resistance.

## Data Availability

All the data generated or analyzed during this study are included in this published article and its Supplementary Information Files.

## Supplementary Materials

The following supporting information can be downloaded at: https://www.mdpi.com/article/10.3390/plants15162421/s1, Figure S1: Ka/Ks ratios analysis for selective pressure acting on apple Ca^2+^-ATPase genes; Figure S2: Identification of conserved motifs within Ca^2+^-ATPase genes using MEME analysis; Figure S3: Analysis of *cis*-acting elements associated with apple Ca^2+^-ATPase genes; Figure S4: Heatmap of the relative synonymous codon usage (RSCU) for the protein sequences within the Ca^2+^-ATPase gene family in apple; Figure S5: The correlation matrix illustrating the relationships among various codon usage parameters; Figure S6: Analysis of the expression patterns of the Ca^2+^-ATPase gene family in response to infection with *A. alternata*; Figure S7: Analysis of the expression patterns of the Ca^2+^-ATPase gene family following treatment with CaCl_2_, SA, and MeJA; Table S1: Analysis of the physicochemical properties of the apple Ca^2+^-ATPase gene family; Table S2: Prediction of subcellular localization of apple Ca^2+^-ATPase proteins; Table S3: Secondary structure analysis of apple Ca^2+^-ATPase gene family proteins; Table S4: Primer sequences used for cloning and vector construction; Table S5: Primer sequences used for qRT-PCR in *Malus domestica*.
