# Supplementary material for: Genome-Wide Identification of the Ca2+-ATPase Gene Family and Functional Analysis of MdACA39 in Resistance to Alternaria alternata in Malus domestica
Source: Plants (Basel). 2026 Aug 8;15(16):2421. doi: 10.3390/plants15162421 (PMC13516274; doi:10.3390/plants15162421)
Supplement: Supplementary file 1 [file plants-15-02421-s001.zip › plants-4469881-supplementary.pdf]

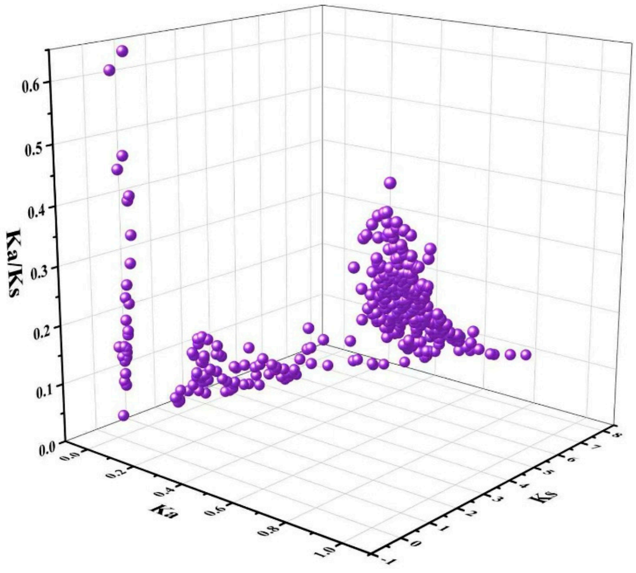

Figure S1. Ka/Ks ratios analysis for selective pressure acting on apple Ca^{2+}-ATPase genes.

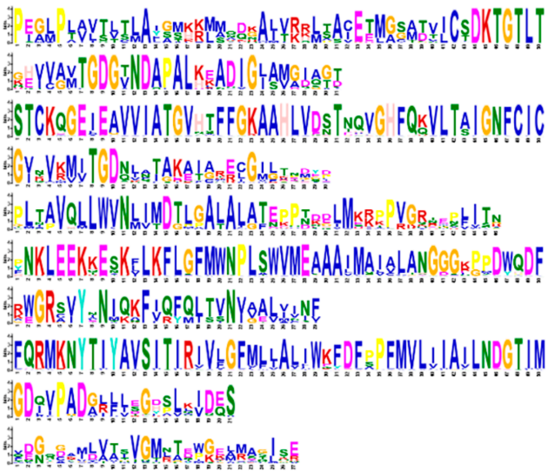

	Logo	Name	E-value	Sites	Width
1.		PEGLPLAVTLTLAIGMKKMMQDKAJVRRLTACETMGSATVJCSDKTGTLT	4.8e-1544	45	50
2.		GHVVAVTGDGTNDAPALKEADIGJAMGIAGT	6.3e-947	44	31
3.		STCKQGEIEAVVIATGVHTFFGKAAHLVDSTNQVGHFQKVLTAIGNFCIC	2.6e-548	14	50
4.		GVNVKMITGDNLATAKAIARECGILTNDYD	4.9e-657	43	30
5.		PLTAVQLLWVNLIMDTLGALALATEPPTDDLMKRPPVGRTEPLITN	7.9e-793	28	46
6.		PNKLEEKKESKFLKFLGFMWNPLSWVMEAAAIMAIALANGGGKPPDWQDF	5.4e-499	14	50
7.		RWGRSVYNNIQKFIQFQLTVNVAALVINF	2.6e-493	27	29
8.		FQRMKNYTIYAVSITIRIVLGFMLLALIWKFDFPPFMVLIIAILNDGTIM	2.9e-492	13	50
9.		GDQVPADGLLLEGDSLKIDZS	1.7e-454	39	21
10.		VDGQGAMLVTSVGMNTEWGELMAGISE	3.6e-480	45	27

Figure S2. Identification of conserved motifs within Ca^{2+}-ATPase genes using MEME analysis. Sequence logo representation of the conserved motifs, where the total height of each stack reflects the conservation level at each position, and the height of individual letters within the stack corresponds to the relative frequency of specific amino acids. Amino acid sequences name of conserved motifs identified in Ca^{2+}-ATPase proteins. The associated combined *E*-values, and motif lengths are presented on the left side of the figure.

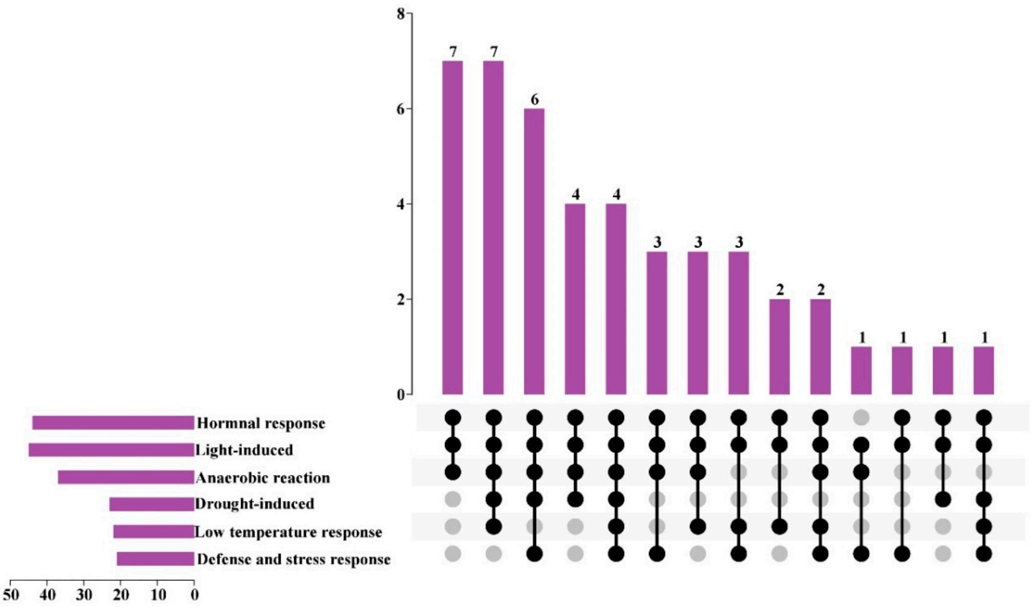

Figure S3. Analysis of *cis*-acting elements associated with apple Ca^{2+}-ATPase genes. The left y-axis indicates the number of Ca^{2+}-ATPase genes responsive to each cis-acting element. The right y-axis denotes the number of Ca^{2+}-ATPase genes categorized according to specific *cis*-acting element groups. The dot plot located at the lower right illustrates the distribution of the various *cis*-acting elements examined.

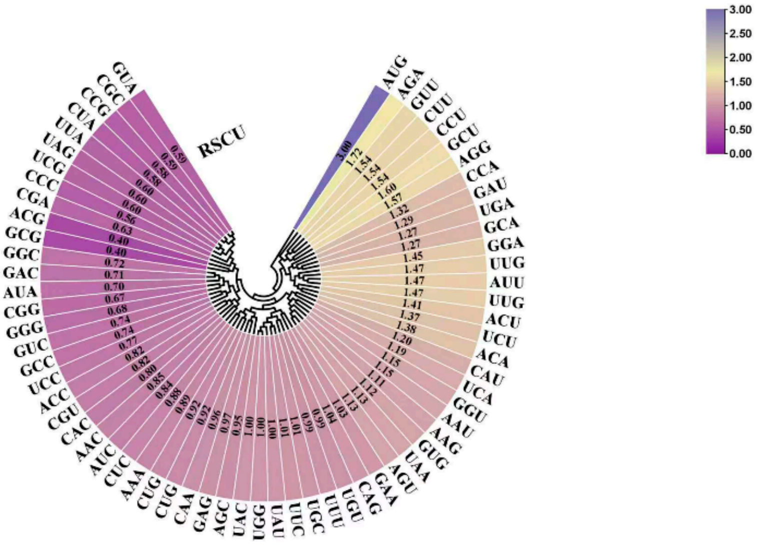

Figure S4. Heatmap of the relative synonymous codon usage (RSCU) for the protein sequences within the Ca^{2+}-ATPase gene family in apple. The outer ring denotes the codons corresponding to specific amino acids, while the inner ring illustrates the RSCU values associated with each codon.

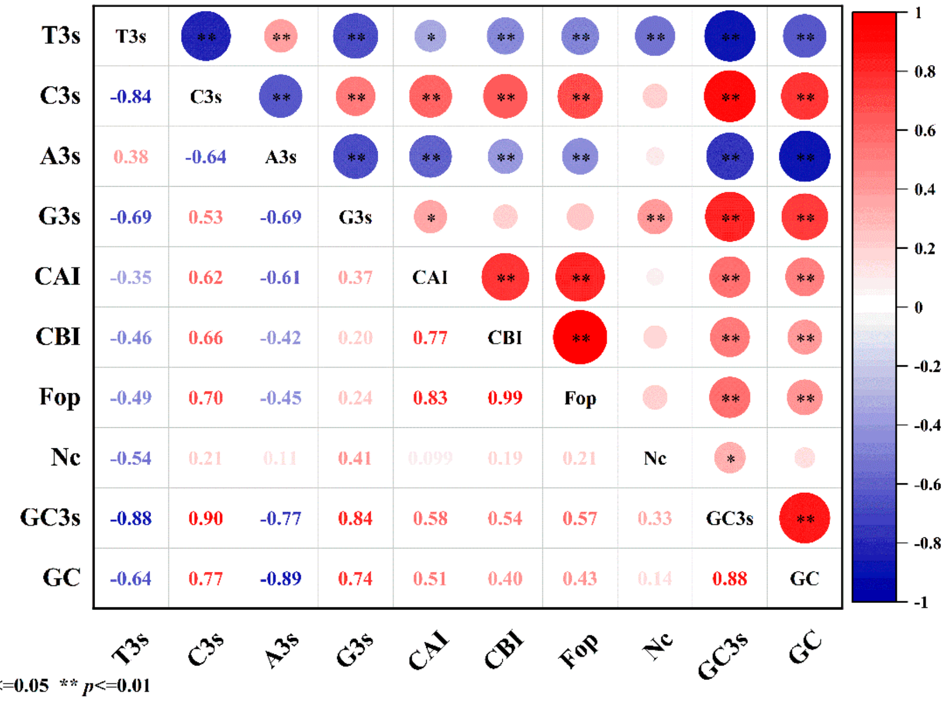

Figure S5. The correlation matrix illustrating the relationships among various codon usage parameters. Within the matrix, the magnitude of the circles and the intensity of the coloration correspond to the strength of the correlations, where red signifies positive correlations, blue denotes negative correlations, and white indicates the absence of correlation, and larger circles together with stronger color saturation correspond to higher correlation coefficients. Mean difference > $LSD_{0.01}$ (0.0227), > $LSD_{0.05}$ (0.0172).

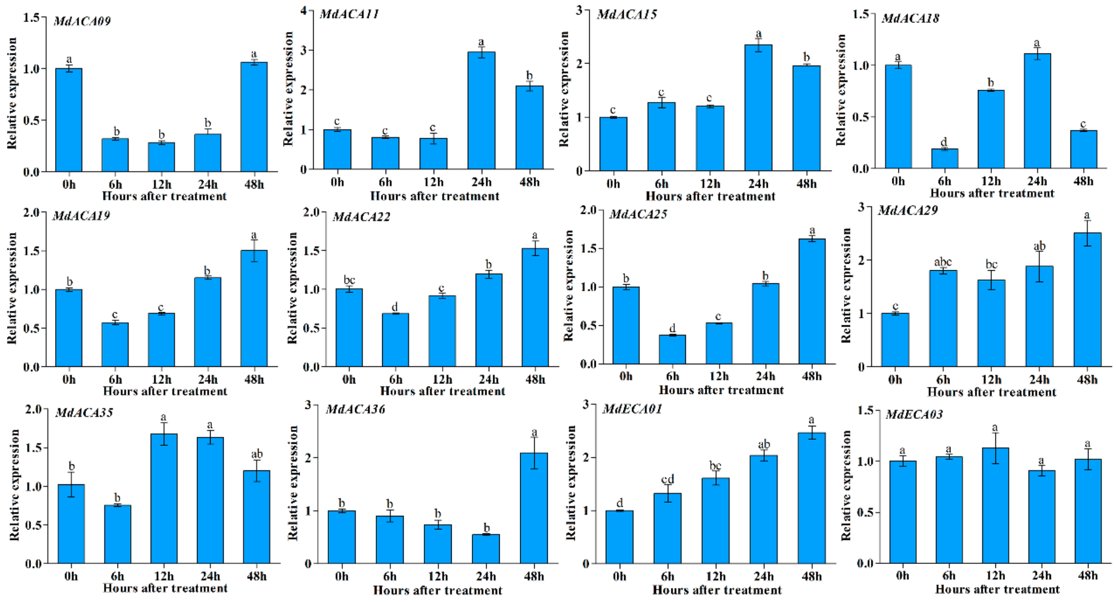

Figure S6. Analysis of the expression patterns of the Ca^{2+}-ATPase gene family in response to infection with *A. alternata*. The y-axis denotes relative gene expression levels, while the x-axis corresponds to the time points assessed (0, 6, 12, 24, and 48 h). Statistical significance was determined at the 0.05 level, with differing lowercase letters indicating significant differences between time points, whereas identical lowercase letters denote the absence of statistically significant differences.

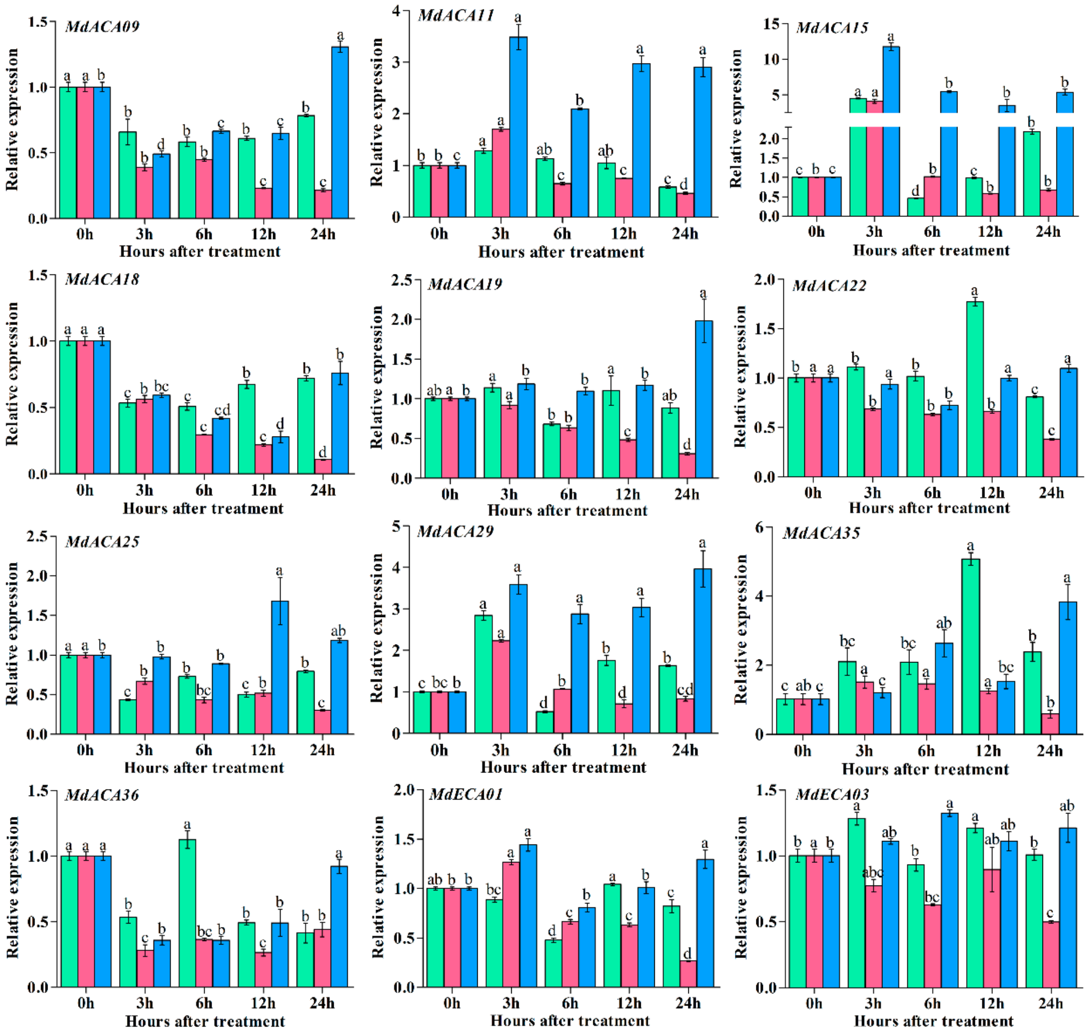

Figure S7. Analysis of the expression patterns of the Ca^{2+}-ATPase gene family following treatment with $CaCl_2$, SA, and MeJA. The y-axis denotes relative gene expression levels, while the x-axis corresponds to the time points assessed (0, 3, 6, 12, and 24 h). Statistical significance was determined at the 0.05 level, with differing lowercase letters indicating significant differences between time points, whereas identical lowercase letters denote the absence of statistically significant differences.

Table S1. Analysis of the physicochemical properties of the apple Ca^{2+}-ATPase gene family

Gene name	Gene accession	GDR	amino acid/aa	Molecular weight/kDa	pI	Instability index	Aliphatic index	GRAVY
MdACA01	MD00G1145700	MDP0000254558	943	105.79	6.21	39.90	103.29	0.12
MdACA02	MD02G1011600	MDP0000162032	947	103.68	6.05	34.82	101.44	0.09
MdACA03	MD02G1185000	-	1015	110.66	5.74	34.73	99.48	0.18
MdACA04	MD02G1216000	MDP0000152620	991	108.87	7.06	34.10	101.40	0.08
MdACA05	MD02G1298100	-	1108	121.57	6.54	40.23	102.46	0.14
MdACA06	MD03G1197800	MDP0000277881	485	52.43	5.31	34.84	104.39	0.14
MdACA07	MD04G1058300	MDP0000278361	973	106.86	6.25	35.45	107.07	0.17
MdACA08	MD04G1223800	MDP0000120301	1031	113.75	8.31	34.56	101.31	0.03
MdACA09	MD06G1186400	MDP0000157578	956	105.26	5.99	38.33	105.33	0.12
MdACA10	MD07G1026000	-	1041	113.78	6.08	37.38	104.65	0.14
MdACA11	MD08G1085200	MDP0000201907	1016	110.87	6.39	35.55	102.53	0.18
MdACA12	MD08G1085600	MDP0000297953	1166	127.46	6.64	33.82	100.28	0.19
MdACA13	MD08G1130200	MDP0000181085	954	105.03	7.24	34.55	101.45	0.04
MdACA14	MD09G1160300	MDP0000121822	1100	120.23	7.54	35.61	102.09	0.06
MdACA15	MD09G1171300	MDP0000303799	948	104.45	5.50	36.68	107.10	0.15
MdACA16	MD09G1223600	MDP0000249645	954	104.84	6.17	32.68	103.14	0.07
MdACA17	MD11G1278100	MDP0000506038	1155	128.10	6.39	35.90	96.70	-0.05
MdACA18	MD12G1080700	MDP0000283858	1039	113.47	5.69	33.54	110.19	0.22
MdACA19	MD12G1080800	MDP0000283858	1450	160.58	6.71	36.73	99.29	-0.04
MdACA20	MD12G1240600	MDP0000846195	1033	113.72	8.55	35.05	101.62	0.03
MdACA21	MD13G1201600	MDP0000170317	1012	112.20	7.75	31.42	94.75	0.02
MdACA22	MD13G1241000	MDP0000230664	1070	117.02	6.92	37.22	103.85	0.07
MdACA23	MD13G1247200	MDP0000259837	955	105.21	6.51	36.47	101.27	0.09
MdACA24	MD14G1077000	MDP0000319861	1039	113.23	6.04	34.24	108.96	0.20
MdACA25	MD14G1192600	MDP0000266132	956	105.18	6.10	38.51	104.62	0.11
MdACA26	MD15G1070800	-	1016	110.71	6.31	37.43	102.44	0.19
MdACA27	MD15G1108400	MDP0000136397	1032	113.75	6.86	40.21	100.40	0.02
MdACA28	MD15G1296400	-	1015	110.90	5.75	35.29	100.24	0.18
MdACA29	MD15G1317200	MDP0000319016	942	105.89	6.09	37.63	104.01	0.13
MdACA30	MD15G1425900	MDP0000484742	1028	113.28	8.34	31.28	97.45	0.04
MdACA31	MD15G1426200	MDP0000581549	1028	113.15	7.50	31.63	99.35	0.05
MdACA32	MD15G1429900	MDP0000905145	965	106.07	6.07	29.77	99.16	0.08
MdACA33	MD16G1201800	MDP0000495793	1027	113.36	8.46	29.95	97.08	0.04
MdACA34	MD16G1245600	MDP0000260974	2062	224.01	7.67	33.98	106.09	0.14
MdACA35	MD16G1246100	MDP0000198731	868	93.70	5.86	38.58	99.87	0.01
MdACA36	MD16G1254200	MDP0000150049	955	105.01	6.13	36.11	103.11	0.09
MdACA37	MD17G1046600	MDP0000810883	956	105.31	6.45	35.64	104.01	0.12
MdACA38	MD17G1146100	MDP0000167866	1103	120.13	6.94	37.20	103.84	0.10
MdACA39	MD17G1155800	MDP0000290422	950	104.38	5.37	38.08	106.37	0.15
MdECA01	MD04G1116400	MDP0000159130	1063	116.26	5.30	35.91	99.13	0.04
MdECA02	MD07G1154700	MDP0000764137	1050	115.85	5.79	37.07	98.20	0.11
MdECA03	MD08G1080200	MDP0000305742	1002	109.71	6.04	40.36	103.43	0.23
MdECA04	MD11G1117700	MDP0000282919	1078	118.21	5.31	36.25	99.75	0.04
MdECA05	MD12G1132100	MDP0000401049	1063	116.41	5.50	34.34	98.20	0.04
MdECA06	MD15G1067300	MDP0000230529	1002	109.69	5.84	38.65	102.46	0.23

Table S2. Prediction of subcellular localization of apple Ca^{2+}-ATPase proteins

Protein name	Cytoplasm	Chloroplast	Plasma membrane	Vacuole	Golgi Apparatus	Endoplasmic Reticulum
MdACA01	-	-	13	-	-	1
MdACA02	-	-	12	1	-	1
MdACA03	-	-	11	-	-	3
MdACA04	1	-	10	3	-	-
MdACA05	-	1	13	-	-	1
MdACA06	1	-	10	2	-	1
MdACA07	1	-	12	2	-	-
MdACA08	-	1	13	-	-	-
MdACA09	1	-	12	1	-	-
MdACA10	-	-	9	2	2	1
MdACA11	-	-	11	-	-	3
MdACA12	-	-	10	1	2	1
MdACA13	-	-	13	-	-	1
MdACA14	-	-	13	-	-	1
MdACA15	-	-	12	2	-	-
MdACA16	1	-	13	-	-	-
MdACA17	-	-	10	1	2	1
MdACA18	-	-	10	3	-	1
MdACA19	-	-	11	-	-	3
MdACA20	-	-	12	-	-	2
MdACA21	-	-	14	-	-	-
MdACA22	-	-	12	-	-	1
MdACA23	1	-	12	1	-	-
MdACA24	-	-	10	2	1	1
MdACA25	1	-	12	1	-	-
MdACA26	-	-	11	-	-	3
MdACA27	-	1	12	-	-	1
MdACA28	-	-	11	-	-	3
MdACA29	-	-	12	-	-	2
MdACA30	-	-	11	2	-	1
MdACA31	1	-	9	2	-	2
MdACA32	-	-	11	-	1	2
MdACA33	1	-	9	1	-	2
MdACA34	-	-	11	-	2	1
MdACA35	-	-	9	1	-	4
MdACA36	-	-	12	1	1	-
MdACA37	1	-	11	2	-	-
MdACA38	-	-	13	-	-	1
MdACA39	-		12	-	2	-
MdECA01	2	-	9	-	-	3
MdECA02	-	-	10	-	1	3
MdECA03	-	-	9	2	-	3
MdECA04	-	-	10	1	-	3
MdECA05	2	-	9	-	-	3
MdECA06	-	-	10	2	-	2

Note: "-" indicates the lack of expression of Ca^{2+}-ATPase family members in the specified organelle, whereas numerical values correspond to the expression levels observed within each organelle.

Table S3. Secondary structure analysis of apple Ca^{2+}-ATPase gene family proteins

Protein	Alpha helix/%	Extended strand/%	Random coil/%	Beta turn/ %
MdACA01	47.83	16.54	6.04	29.59
MdACA02	50.69	15.84	5.70	27.77
MdACA03	44.83	18.92	7.29	28.97
MdACA04	48.64	15.94	5.35	30.07
MdACA05	43.86	19.13	6.77	30.23
MdACA06	49.48	17.73	6.80	25.98
MdACA07	49.95	15.31	5.65	29.09
MdACA08	43.06	19.11	6.60	31.23
MdACA09	47.91	17.47	6.07	28.56
MdACA10	43.90	18.64	6.82	30.64
MdACA11	43.90	19.19	7.38	29.53
MdACA12	41.25	20.84	7.20	30.70
MdACA13	49.58	16.46	5.66	28.30
MdACA14	41.18	19.00	6.55	33.27
MdACA15	48.21	16.88	5.80	29.11
MdACA16	49.27	16.25	5.14	29.35
MdACA17	43.20	18.87	5.89	32.03
MdACA18	43.60	18.29	6.35	31.76
MdACA19	42.41	18.62	7.59	31.38
MdACA20	42.69	19.46	6.10	31.75
MdACA21	45.45	18.48	6.13	29.94
MdACA22	42.80	19.44	6.17	31.59
MdACA23	50.79	15.50	4.82	28.90
MdACA24	45.14	17.71	6.74	30.41
MdACA25	47.38	17.05	6.17	29.39
MdACA26	44.09	18.80	7.38	29.72
MdACA27	46.41	16.09	5.23	32.27
MdACA28	42.76	19.01	7.29	30.94
MdACA29	46.50	16.03	5.84	31.63
MdACA30	44.94	19.65	6.23	29.18
MdACA31	43.77	19.16	6.32	30.74
MdACA32	44.35	20.31	6.32	29.02
MdACA33	43.14	19.67	6.52	30.67
MdACA34	36.91	22.60	8.10	32.40
MdACA35	40.09	19.59	7.37	32.95
MdACA36	49.11	15.39	5.24	30.26
MdACA37	49.06	16.53	5.44	28.97
MdACA38	41.89	18.50	6.62	33.00
MdACA39	47.37	17.05	5.58	30.00
MdECA01	44.87	17.50	6.21	31.42
MdECA02	44.95	17.71	5.90	31.43
MdECA03	46.21	17.76	5.79	30.24
MdECA04	44.81	17.63	5.75	31.82
MdECA05	44.31	18.06	6.21	31.42
MdECA06	46.61	17.86	5.29	30.24

Table S4. Primer sequences used for cloning and vector construction.

Genes	Vector	Sequences (5′-3′)	
		Forward primer	Reverse primer
MdACA39		ATGGCTGAAGATCTGGAGAAACCAT	GACTGTGTGGGCTGATTGAATGGCA
MdACA39	pCAMBIA2300-OE	GTCGACATGGCTGAAGATCTGGAGAAACC (*Sal*I site is underlined)	AGGCCTTCAGACTGTGTGGGCTGATT (*Stu*I site is underlined)
MdACA39	pCAMBIA1302	AGATCTATGGCTGAAGATCTGGAGAAACC (*Bgl*II site is underlined)	ACTAGTGACTGTGTGGGCTGATTGAATGGCA (*Spe*I site is underlined)
PDK intron	pCAMBIA2300-PDK intron	GTCGACCGCTCGAGGAATTCGGTACCCCAAT (*Sal*I site is underlined)	AGGCCTGATCCAAGCTTATCGATTTCGAACC (*Stu*I site is underlined)
mdaca39	pCAMBIA2300-mdaca39-PDK intron	CTGCAGTCCCCGAAGCCTGATAGTTGGAAGC (*Pst*I site is underlined)	AGGCCTCACCACGAAAGCGCACATCAACATA (*Sal*I site is underlined)
mdaca39	pCAMBIA2300-RNAi	AGGCCTCACCACGAAAGCGCACATCAACATA (*Stu*I site is underlined)	TCTAGATCCCCGAAGCCTGATAGTTGGAAGC (X*ba*I site is underlined)

Table S5. Primer sequences used for qRT-PCR in *Malus domestica*.

Gene	Forward primer	Reverse primer
MdACA09	CTCTTGCAAATGGAGGGGGT	TCACCGGGTACAAGAACAGC
MdACA15	AGCCAGGGGTGGTCATTTCT	ATTCCAGGCATCTCCGGTC
MdACA18	TCGTGGATCTATTGAAAAGCTTGGT	TTCGGGGAGCTTTCTCCTTGT
MdACA19	GAGAGCTTTCTCCGGTCGAG	GATTTTGTGCGGCGTCCATC
MdACA22	TCGTGGCAGCCATTTCATCT	TCCTCGGAAGGTGAGGACAA
MdACA23	GTGTTTGCAAAGGGTGTGGAG	TTGCAGAGGCAGAGGTCCAA
MdACA25	CTCTTGCAAATGGAGGGGGT	CCAGGAACTAGAACCGAAGCA
MdACA29	TGAGGATGGTGTCCGCAAAA	CCATGTCCCGTATCGCAGTT
MdACA35	GTCATTGTTGTCACAGCCGTT	TTGAGGCTCACAATCTTGCTC
MdACA36	ATGCTTCTACTCGGTGCTTTCT	AAGTTGAGCCACGCCTTTCC
MdACA39	TCCCCGAAGCCTGATAGTTG	CACCACGAAAGCGCACATC
MdECA01	GATGGGGAAGAAGGCGGTG	ATATCAGCGGGCACCTTATCC
MdECA03	ACCACTAGTATCCGGGCTGA	GCTGTTGACTTGTTGTCCCC
MdPR1	GCAGCAGTAGGCGTTGGTCCCT	CCAGTGCTCATGGCAAGGTTTT
MdPR2	TGGTGATCAAGCAACCCCTC	TCTCAACTGGTTTACCGTCCT
MdMYC2	CTGGACCTACGCCATCTTCT	CTTGCGGTACTCTTGTTCGG
MdPR4	GGGACAAAGTGCGACGAATG	GCTCCAGTCCGAGTGTTTGT
MdWRKY33	CGGACAGAAACGGGACTGAAA	TTCCTTGCTGGGTGTCCTTAC
MdCML	TCAAGCACAGAATGGGAGAGG	TTCTTCATCCGCGTCGTTTTC
MdCDPK4	GCCATTTTGGGCAGAAACCC	TCATGTGCAGTTAAGCGGTCT
Alta1	TCCGAATTCTACGGACGCAA	TGAGCAGAGCAGGTGAAGTC
MdSOD	GTTGACAAGCAGATTCCTC	CCAATAATACCGCAAGCC
MdPOD	TTGACTGTACGAGGATACG	TGTTGGAGCAGCATAGTT
MdCAT	CTCATCACAACAATCACCAT	TCTCAATGACGCACTTCT
MdAPX	TGGACTCCCAACCCCCTTAT	AAAGCATCTTCATCCGCAGC
MdGPX	AGCTCTGCAACTCCCGAAAT	GCTGATTGCGAAGACCCAAG
Mdtubulin	AGGATGCTACAGCCGATGAG	GCCGAAGAACTGACGAGAATC
